# Efficiency, accuracy and robustness of probability generating function based parameter inference method for stochastic biochemical reactions

**DOI:** 10.1371/journal.pcbi.1014160

**Published:** 2026-04-10

**Authors:** Shiyue Li, Yiling Wang, Zhanpeng Shu, Ramon Grima, Qingchao Jiang, Zhixing Cao

**Affiliations:** 1 State Key Laboratory of Bioreactor Engineering, East China University of Science and Technology, Shanghai, China; 2 College of Electrical Engineering, Shanghai Dianji University, Shanghai, China; 3 School of Biological Sciences, University of Edinburgh, Edinburgh, United Kingdom; 4 Department of Chemical Engineering, Queen’s University, Kingston, Canada; Universidade de Vigo, SPAIN

## Abstract

Biochemical reactions are inherently stochastic, with their kinetics commonly described by chemical master equations (CMEs). However, the discrete nature of molecular states renders likelihood-based parameter inference from CMEs computationally intensive. Here, we introduce an inference method that leverages analytical solutions in the probability generating function (PGF) space and systematically evaluate its efficiency, accuracy, and robustness. Across both steady-state and time-resolved count data, our numerical experiments demonstrate that the PGF-based method consistently outperforms existing approaches in terms of both computational efficiency and inference accuracy, even under data contamination. These favorable properties further enable the extension of the PGF-based framework to model selection—a task typically considered computationally prohibitive. Using time-resolved data, we show that the method can correctly identify complex gene expression models with more than three gene states, a task that cannot be reliably achieved using steady-state data alone.

## Author summary

Biochemical processes within cells, such as gene expression, are inherently stochastic. To understand these dynamics, researchers use mathematical models like the Chemical Master Equation (CME) to infer kinetic parameters from experimental data. However, traditional inference methods often face a bottleneck: they are either computationally too slow or lack the necessary accuracy when dealing with the complex, noisy data produced by modern single-cell experiments. In this study, we introduce a high-performance inference framework based on the Probability Generating Function (PGF). By leveraging analytical solutions, our method achieves exceptional efficiency and accuracy across both steady-state snapshots and transient, time-resolved data. We demonstrate that the PGF-based approach is highly robust, maintaining reliable performance even when data is corrupted by experimental artifacts such as molecular loss or extreme outliers. Crucially, we extend this framework to the critical task of model selection. Using a cross-validation strategy, our method can accurately distinguish between competing biological hypotheses—for instance, correctly identifying the number of hidden states a gene transitions through before activation. This versatile and scalable tool provides a powerful resource for researchers to decode the hidden mechanisms of life from complex single-cell datasets.

## Introduction

Biochemical reactions are inherently stochastic, arising from the random collisions of biomolecules, whose movements are naturally unpredictable. Gene expression is a quintessential example of this phenomenon, with extensive experimental evidence confirming its stochasticity [1–5]. For clarity, we will primarily use gene expression to illustrate our proposed method, though the approach is generalizable. The stochastic nature of these reactions necessitates a probabilistic framework for quantitative kinetic analysis, enabling a more precise understanding of molecular-level processes [6,7].

A biochemical reaction system can be generally represented by a set of reaction equations [8,9]:


$$\sum_{i}s_{ir}X_{i}\overset{k_{r}\;}{\to }\sum_{i}s_{ir}^{\prime }X_{i},\;r=1,\cdots ,R,$$ (1)

where *s*_*ir*_ and *s*_*ir*_′ are the stoichiometric coefficients of species *X*_*i*_ in reaction *r*. Assuming the law of mass action, the rate of reaction *r* is given by


$$f_{r}(𝐧)=k_{r}\Omega \prod_{i}{\left(\frac{n_{i}}{\Omega }\right)}^{s_{ir}},$$ (2)

where *k*_*r*_ is the rate constant, $𝐧=[n_{1},\cdots ,n_{N}{]}^{⊤}$, *n*_*i*_ is the molecule count of species *X*_*i*_, and Ω is the reaction volume. A fundamental task in analyzing the kinetics of the reaction system in Eq. (1) is inferring the kinetic parameters *k*_*r*_ from observed molecule counts $(n_{i})\in \mathbb{N}$ of certain species—a process known as parameter inference or estimation in systems biology [10–12], or system identification in control theory [13,14]. ℕ is the set of natural numbers.

Parameter inference is fundamentally an inverse problem that necessitates repeated forward computations of the kinetic model. Given the various approaches available for kinetic model computation, the inference methods in the literature can be broadly classified into four groups. **The first group** employs maximum likelihood estimation (MLE) combined with finite state projection (FSP) [11,15–17]. FSP solves a set of chemical master equations (CMEs) [9,18], which are difference-differential equations commonly used to describe stochastic reaction kinetics. This approach assumes that the probability of molecule counts exceeding a certain threshold (truncated size) is zero [19,20]. However, the computational efficiency of these methods declines rapidly as the number of species, and consequently the number of equations, increases exponentially. Moreover, the selection of the truncated size requires careful consideration to achieve an intricate balance between computation load and precision. **The second group** employs the method of moments (MOM), where a few low-order moments are calculated both from the molecule count data and the kinetic models, and then used to generate a Gaussian-like synthetic likelihood for inference [12,21–24]. These methods are computationally efficient, requiring the solution of only a few differential equations. However, their accuracy can be unsatisfactory, especially when higher-order moments are needed to derive a sufficient number of moment equations for inference. In such cases, the accuracy of moments computed from small sample sizes can be compromised [10]. Additionally, if the reaction involves multiple reactant molecules (i.e., it is not a first-order reaction), denoted by $\sum_{i}s_{ir}>1$, the moment equations derived from the corresponding CMEs are not closed, necessitating the use of various moment closure methods [18,25,26]. Moment closure is inherently an approximation, potentially introducing another layer of inaccuracy. **The third group** employs an Approximate Bayesian Computation (ABC) scheme combined with the Stochastic Simulation Algorithm (SSA) for parameter inference [27–29]. ABC approximates the posterior distribution by simulating data under various parameter values and comparing it to observed data. Parameters yielding simulations that closely match the observed data are accepted as approximations of the true posterior. This approach is advantageous as it bypasses explicit likelihood calculations, with SSA providing an exact method for generating simulation data. However, this framework has drawbacks, including the need for large simulation samples to accurately approximate the posterior, which can be computationally expensive, and sensitivity to tuning parameters such as the tolerance level and distance metric.

The final group is the PGF-based inference method [30–32], which we systematically investigate in this work. This method computes the empirical PGF directly from count data and compares it with the analytical PGF solution derived from the model, using either the density power divergence [30,31] or the mean squared error [32] as the objective function. Minimizing this discrepancy yields the inferred kinetic parameters. Ref. [32] has demonstrated several advantages of the PGF-based inference method: (i) Analytical PGF solutions are available for a broad class of gene expression models. Traditionally, these solutions have been used by performing Taylor expansions to recover probability mass functions, followed by maximum likelihood estimation (MLE) for parameter inference. However, this approach is numerically demanding—particularly because PGF solutions often involve hypergeometric functions that require high-order derivatives, which are computationally unstable and require high numerical precision. As a result, such methods are not widely adopted [33,34]. In contrast, the PGF-based method circumvents the need for differentiation by directly evaluating the PGF over a range of variable values, thereby improving both stability and computational efficiency. This approach enables full utilization of existing PGF solutions. (ii) The PGF-based method achieves computational efficiency comparable to MOM, while maintaining inference accuracy on par with MLE. Building on these advantages, we systematically evaluate the accuracy, efficiency, and robustness of the PGF-based method under two types of data contamination: binomial downsampling and outliers. Furthermore, we extend the PGF-based framework in Ref. [32] from steady-state to time-resolved count data. Within this extended setting, we develop a model selection strategy based on cross-validation. Using this approach, we demonstrate that time-resolved data enables reliable identification of complex gene expression models with more than three gene states—a task that cannot be accomplished using steady-state data alone.

Section Results I presents the PGF-based inference method for steady-state count data. Section Results II evaluates its computational efficiency, accuracy, and robustness, with a particular focus on the sensitivity of parameter estimates in the presence of technical noise (downsampling) and data outliers. Section Results III extends the method to time-resolved count data, and Section Results IV develops a model-selection framework based on PGF inference. Section Discussion concludes the paper and outlines future research directions.

## Results

### PGF-based inference method for steady-state count data

Consider a reaction system consisting of *N* species (*X*_*i*_ for i=1,⋯,N) and *R* reactions as defined by Eq. (1) with reaction rates given by Eq. (2). The kinetics of this system can be effectively described using the probabilistic framework of CMEs


$$\frac{\text{d}}{\text{d}t}P(𝐧,t)=\sum_{r=1}^{R}({𝔼}^{-{𝐬}_{r}}-1)f_{r}(𝐧)P(𝐧,t)$$ (3)

Where *P*(**n**,*t*) represents the probability of observing *n*_*i*_ copies of molecule *X*_*i*_ for i=1,⋯,N in the system at time *t*. The vector **s**_*r*_ is defined as


$${𝐬}_{r}=[{\bar{s}}_{1r},\;{\bar{s}}_{2r},\;\cdots ,\;{\bar{s}}_{Nr}{]}^{⊤}$$

with ${\bar{s}}_{ir}=s_{ir}^{\prime }-s_{ir}$. The step operator ${𝔼}^{-{𝐬}_{r}}$ acts on a general function $f(n_{1},\cdots ,n_{N})$ as follows


$${𝔼}^{-{𝐬}_{r}}f(n_{1},\cdots ,n_{N})=f(n_{1}-{\bar{s}}_{1r},\cdots ,n_{N}-{\bar{s}}_{Nr}).$$

This indicates that applying the operator shifts the arguments of the function *f* by subtracting the corresponding components of the vector **s**_*r*_. Solving Eq. (3) is challenging due to the presence of both discrete variables (*n*_*i*_, which are integers) and continuous variables (*t*). The PGF method offers a way to circumvent this challenge. The PGF is defined as


$$G(𝐳,t)=\left\langle \prod_{i=1}^{N}z_{i}^{n_{i}}\right\rangle =\sum_{n_{1},\cdots ,n_{N}}P(𝐧,t)\prod_{i=1}^{N}z_{i}^{n_{i}}$$ (4)

in which $𝐳=[z_{1},\cdots ,z_{N}{]}^{⊤}$ and ⟨·⟩ is the expectation operator. Essentially, the PGF provides a compact way to represent the full count distribution *P*(**n**,*t*) without listing the probability of every possible count vector explici*t*ly. It is defined as the *z*-transform of the probability mass function *P*(**n**,*t*), which encodes all probabilities into a single analytic function of an auxiliary variable (or vector) *z*. In this sense, the *z*-*t*ransform plays a role for discrete random variables analogous to that of the Laplace transform for continuous variables, and it is widely used because moments and other distributional properties can be extracted directly from the transformed function.

By applying Eqs. (4), (3) can be conveniently transformed into a set of partial differential equations (PDEs). These resulting PDEs can then be tackled using various standard methods for solving PDEs. This approach, known as the PGF method, has been effectively employed to solve a wide range of kinetic models, as summarized in Table A in S1 Text. In Section A in S1 Text, we also introduce some properties of the PGF, which allow the construction of the PGF for more complex systems by using the solutions in Table A in S1 Text as foundational building blocks [25,32,35–43].

Building on the PGF solutions of various kinetic models, we now introduce the PGF-based inference method for the steady-state distribution.

Consider a population of *n*_*c*_ cells where the count of the *j*-th species in the *i*-th cell is *n*_*ij*_ for $i=1,\cdots ,n_{c}$ and j=1,⋯,N. Following Eq. (4), the joint empirical PGF (EPGF) for this count data is given by


$$G(𝐳)=\frac{1}{n_{c}}\sum_{i=1}^{n_{c}}\prod_{j=1}^{N}z_{j}^{n_{ij}}.$$ (5)

Moreover, from the kinetic model of interest we can derive a PGF, denoted by ${𝒢}_{\theta }(𝐳)$, where θ denotes the kinetic parameters. The inference task is then to estimate θ by minimizing the discrepancy between *G*(**z**) and ${𝒢}_{\theta }(𝐳)$ under a chosen metric. Here, we adopt the mean squared error, defined as


$$J(\theta )=\int_{\Gamma }g_{\theta }(𝐳)\;\text{d}𝐳,$$ (6)

where


$$g_{\theta }(𝐳)=\|G(𝐳)-{𝒢}_{\theta }(𝐳){\|}_{2}^{2},$$

and $\Gamma =[z_{\text{min}},z_{\text{max}}{]}^{N}$.

It is worth noting that the mean squared error formulation of $g_{\theta }(𝐳)$ is a special case of the density power divergence with hyperparameter α=1 (see Eq. (2.1) in Ref. [30]), and that the density power divergence approaches the Kullback–Leibler divergence as α→0 [31]. The kinetic parameters are estimated by solving the optimization problem


$$\hat{\theta }=\arg\min_{\theta }J(\theta ).$$ (7)

To reduce computational effort, we apply the Gauss quadrature method to approximate the integral Eq. (6) as follows


$$J(\theta )=a_{z}^{N}\sum_{𝐢\in ℐ}\omega_{𝐢}g_{\theta }({𝐳}_{𝐢})$$ (8)

where $\omega_{𝐢}=\prod_{j=1}^{N}w_{i_{j}}$, and


$${𝐳}_{𝐢}={\left[a_{z}y_{i_{1}}+b_{z},\cdots ,a_{z}y_{i_{N}}+b_{z}\right]}^{⊤},$$

with


$$a_{z}=\frac{z_{\max}-z_{\min}}{2},\;b_{z}=\frac{z_{\max}+z_{\min}}{2}$$

**Algorithm 1 PGF-based inference method for steady-state count data**

**Input:** Number of cells (*n*_*c*_), the count tuples of *N* species $\left\{(n_{i1},\cdots ,n_{iN})\right\}$ for $i=1,\cdots ,n_{c}$, integration bounds $z_{\min}$ and $z_{\max}$

**Output:** Kinetic parameters θ

1:  Generate Gauss quadrature points and weights $y_{i_{j}}$ and $w_{i_{j}}$ by the command gausslegendre

2:  Compute the joint PGF for count data by using Eq. (5)

3:  Initialize the inferred parameters θ

4:  **while** Threshold not reached **do**

5:   Compute the generating function ${𝒢}_{\theta }(𝐳)$ by using the solutions in Table A in S1 Text and the properties (P1)-(P5) in S1 Text

6:   Compute the loss function J(θ) by using Eq. (8)

7:   Employ the Nelder-Mead optimization algorithm to solve Eq. (7) and update the inferred parameters θ

8:  **end while**

9:  **return** Kinetic parameters θ

Here $y_{i_{j}}\in [-1,1]$ for j=1,⋯,N is the *i*_*j*_-th integration point of the Gauss quadrature of order *N*_*y*_, and $w_{i_{j}}$ is the corresponding integral weight obtained using the gausslegendre function in Julia. The vector $𝐢=[i_{1},\cdots ,i_{N}{]}^{⊤}$ is a sequence of the indices with each component $i_{j}=1,\cdots ,N_{y}$ for all *j*, and the set ℐ contains all such index vectors **i**.

Intuitively, the PGF provides a compact representation of the full probabilistic information of the random variables. For example, factorial moments can be obtained from derivatives of the PGF evaluated at $𝐳=[1,\ldots ,1{]}^{⊤}$. More generally, these derivatives can be viewed as local finite-difference information of the PGF around $𝐳=[1,\ldots ,1{]}^{⊤}$. Therefore, when the PGF is sufficiently characterized, parameter identifiability based on the PGF is (in principle) closely related to identifiability based on factorial moments.

The optimization problem in Eq. (7) is solved using the Nelder–Mead algorithm, implemented through the Optim.jl package in Julia. Since all kinetic parameters are positive, we play the trick – optimizing their logarithmic transformations and subsequently exponentiating the results to obtain the inferred values. The PGF-based inference procedure is summarized in Algorithm 1.

**Fig 1:**
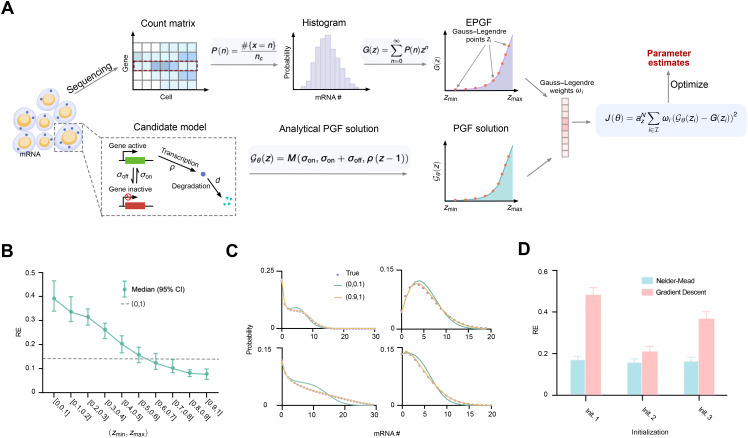
Schematic and performance of the PGF-based inference method. A: Schematic illustration of the PGF-based inference framework for scRNA-seq data using a candidate stochastic gene-expression model (here, the telegraph model). Parameter estimation is performed by minimizing the mismatch between the model’s analytical PGF (e.g., the closed-form solutions listed in Table A of S1 Text; for the telegraph model, the PGF is the Kummer confluent hypergeometric function $M(\sigma_{on},\sigma_{on}+\sigma_{off},\rho (z-1))$) and the empirical PGF, where the mismatch is quantified by Eq. (8). B: Inference accuracy over 200 count distributions generated from randomly sampled kinetic parameters increases as the integration range approaches 1. The best accuracy is achieved at [0.9,1], slightly better than the natural choice [0,1] (dashed line). Bars indicate the 95% confidence interval of relative errors averaged across all three telegraph model parameters. C: Reconstructed distributions from four inferred parameter sets using [0.9,1] (yellow) align more closely with the ground truth (purple dots) than those from [0,0.1] (green). D: The Nelder–Mead algorithm outperforms gradient descent and shows robustness to different initialization strategies.

In Fig 1A, we illustrate the PGF-based inference method using the telegraph model (inset, Fig 1A) [44] and its application to single-cell RNA sequencing (scRNA-seq) data. The scRNA-seq data are typically represented as a gene-by-cell count matrix. For a selected gene, we compute the histogram of its transcript counts and, using Eq. (5), convert this histogram into the EPGF. In the telegraph model, a gene switches between active and inactive states with rates $\sigma_{\text{on}}$ and $\sigma_{\text{off}}$, respectively; transcription occurs only in the active state at rate ρ, and mRNA degrades at rate *d*. The corresponding PGF solution ${𝒢}_{\theta }(z)$ is provided in Table A in S1 Text. The kinetic parameters are $\theta =[\rho ,\;\sigma_{\text{on}},\;\sigma_{\text{off}}{]}^{⊤}$. Under steady-state conditions, the four kinetic parameters cannot be inferred simultaneously; hence, without loss of generality, *d* is set to 1, which is equivalent to normalizing the remaining three parameters by *d*. These parameters are estimated by optimizing the cost function J(θ) in Eq. (6), where the integral is efficiently evaluated using the Gauss quadrature method (Eq. (8)).

Our PGF-based inference method involves two hyperparameters—the integration bounds $z_{\min}$ and $z_{\max}$. To assess their impact on inference accuracy, we uniformly sampled 200 sets of kinetic parameters ρ∈[1,30], $\sigma_{\text{on}}\in [0.01,3]$, and $\sigma_{\text{off}}\in [0.01,10]$. For each set, we generated steady-state count distributions for 1000 cells using the SSA implemented in DelaySSAToolkit.jl [45]. We then performed PGF-based inference with integration ranges $\left[z_{\min},z_{\max}\right]$ varying from [0,0.1] to [0.9,1], along with the natural choice [0,1]. All log-transformed parameters were initialized at 1. As shown in Fig 1B, the inference accuracy, measured by the relative error averaged over all inferred parameters,


$$\text{RE}={\mathrm{Average}}_{i}\;\left(\frac{\left|\theta_{i,\text{true}}-{\hat{\theta }}_{i}\right|}{\theta_{i,\text{true}}}\right),$$

decreases steadily as the integration range approaches 1, reaching its minimum at [0.9,1], which is slightly smaller than that of the natural choice [0,1]. The monotonically decreasing error curve in Fig 1B indicates that inference accuracy is not uniform across the integration range. To better understand this heterogeneity, we selected two extreme ranges from the curve, namely [0,0.1] and [0.9,1], and reconstructed the distributions using the kinetic parameters inferred from each range. The resulting reconstructions are shown in Fig 1C. The reconstruction obtained using [0.9,1] closely matches the ground truth, whereas that obtained using [0,0.1] fails to capture the distribution tail. We ruled out an optimizer artifact by verifying that the obtained solutions satisfied the prescribed optimization tolerance. This behavior is also consistent with the structure of the PGF. Specifically, the PGF is a power series in *z*, and for z∈[0,1], each term *P*(*n*)*z*^n^ decreases with *n*. As *z* becomes smaller, contributions from larger *n* (tail probabilities) decay much faster than those from smaller *n*. Consequently, minimizing the objective in Eq. (8) over small-*z* intervals places disproportionate weight on low-count probabilities and underweights errors in the tail, which can reduce inference accuracy. These results suggest that using an interval near *z* = 1, such as [0.9,1], is a practically effective choice for PGF-based inference and may be broadly useful across a wide range of systems.

As our PGF-based inference method remains optimization-centered, we next investigate how the choice of optimization algorithm and initialization strategy influences inference accuracy. We consider two optimization algorithms—the Nelder–Mead method and gradient descent, the latter representing a broad class of gradient-based methods—and three initialization strategies: (i) setting all log-transformed parameter values to 1; (ii) using log-transformed MOM estimates (see the MOM-based inference method section); and (iii) perturbing the log-transformed MOM estimates by adding random values sampled from 𝒩(1,1.5). Each algorithm–initialization combination was applied to count distributions generated from 200 sets of kinetic parameters, and the relative error was computed for each case. The results, summarized in Fig 1D, show that the Nelder–Mead algorithm consistently outperforms gradient descent across all initialization strategies. Moreover, the inference accuracy of Nelder–Mead remains relatively stable across the three strategies, whereas gradient descent exhibits substantial variation, indicating that Nelder–Mead is less sensitive to initialization. We also found that Nelder–Mead requires less computation time than gradient descent, since it is gradient-free and gradient evaluation in our setting involves additional overhead from hypergeometric functions. Taken together, these results suggest that the optimal configuration for the PGF-based inference method is to use the Nelder–Mead algorithm with the simplest initialization strategy—setting all log-transformed parameter values to 1—together with the integration range [0.9,1].

### Performance evaluation

Given the optimal configuration, we next compare the PGF-based inference method with representative methods from the other three groups of inference methods mentioned in the Introduction – ABC, MOM (see the MOM-based inference method section) and MLE integrated with FSP (see the MLE-based inference method section) from the perspectives of accuracy, computational cost and robustness against data contamination.

**Fig 2:**
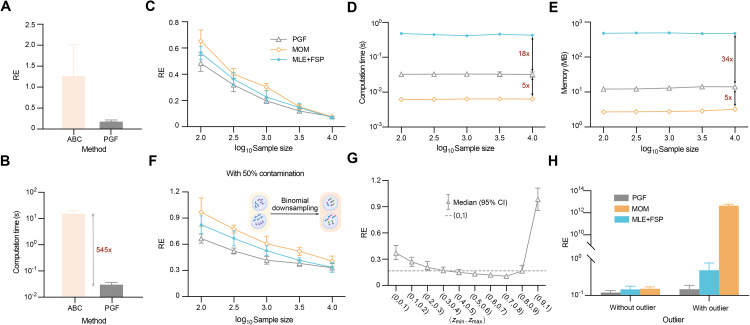
Performance of inference methods in terms of accuracy, efficiency, and robustness. A: Inference accuracy of PGF-based inference and ABC, evaluated by the mean and SEM (error bars) of median REs across 10 replicate datasets for each of five kinetic parameter sets. B: Computational time for PGF-based inference and ABC, showing a > 500-fold speed advantage of the PGF-based method. C: The mean and SEM (error bars) of median REs as a function of sample size for PGF-based inference, MOM, and MLE + FSP. D: Runtime usage for the three methods. E: Memory usage for the three methods. F: PGF-based inference remains the most accurate under binomial downsampling ($x_{i}\sim \text{Binomial}(n_{i},0.5)$), which mimics sequencing capture inefficiency. G: Integration range comparison under outlier contamination, showing that [0,1] achieves the best balance of robustness and accuracy. Error bars indicate the 95% confidence interval of relative errors averaged across all three telegraph model parameters. H: Inference error under moderate outlier contamination (one count of 30 per batch; sample size = 3,000). PGF-based inference is minimally affected, while MOM shows substantial degradation.

To this end, we generated five sets of kinetic parameters for the telegraph model (Table B in S1 Text) and used the SSA to simulate 10 batches of count data for each set, with each batch containing 1000 cells. We first compared the PGF-based inference method with ABC, implemented via ApproxBayes.jl using Gamma(2,2) priors and the default error tolerance ϵ=0.1. For each parameter set, both methods were applied to all batches, and the median of RE was computed to obtain a robust estimate of inference accuracy while mitigating random sampling effects. The mean and SEM (standard error of the mean) of these medians are shown in Fig 2A, demonstrating that the PGF-based method is substantially more accurate than ABC. We also assessed computational efficiency. Both methods were run on a MacBook Air (Apple M2 chip, 16 GB memory), and as shown in Fig 2B, the PGF-based method was over 500 times faster. Due to this large disparity in speed and accuracy, ABC was excluded from further comparisons. Next, we benchmarked PGF-based inference, MOM, and MLE + FSP across a wide range of sample sizes. Using the same five parameter sets and data generation protocol (with varying sample sizes), we generated count data for comparison. For consistency, all methods employed the Nelder–Mead optimizer with hyperparameters g_tol = 10^−20^ and iterations = 2000. As shown in Fig 2C, the averaged RE medians were used to quantify inference error, which decreased with increasing sample size for all methods, as expected. The PGF-based inference method consistently achieved the highest accuracy, with comparable performance from the others only at very large sample sizes ($\sim 10^{4}$). Finally, we evaluated computational time and memory usage (Fig 2D and 2E). MOM was the most efficient, followed by PGF-based inference, while MLE + FSP was 10–100 times more resource-intensive. Considering both accuracy and efficiency, the PGF-based inference method offers the best balance and is the preferred approach.

We next evaluated the robustness of the three inference methods by examining how their accuracy degrades under two types of data contamination: binomial downsampling and outliers. The former simulates the sequencing process, where each transcribed mRNA has a probability of being captured and sequenced. This downsampling effect is commonly modeled by a binomial distribution [46]. To assess its impact, we used the same dataset as in Fig 2C, replacing each count value *n*_*i*_ with a binomial random variable $x_{i}\sim \text{Binomial}(n_{i},0.5)$, representing a 50% chance that each transcript is captured. We then applied the same evaluation protocol as in Fig 2C to compare the three inference methods. As shown in Fig 2F, although inference accuracy degrades for all methods, the PGF-based inference still outperforms the others, with an even larger performance margin. We also examined robustness to outliers by introducing spurious large values into the data to mimic doublets, a common experimental artifact in droplet-based single-cell assays in which two or more cells are encapsulated in the same reaction volume (droplet) and assigned a single barcode. This artifact typically appears as abnormally large count values. Specifically, we contaminated the dataset used in Fig 1B by randomly setting one observation per parameter set to a count of 100, thereby simulating an extreme outlier measurement. We then followed the same evaluation protocol. As shown in Fig 2G, under this contamination, the integration range [0.9,1] is no longer optimal; instead, the natural choice [0,1] becomes nearly optimal. Taken together with the results in Fig 1B, these findings indicate that the integration range [0,1] provides the best balance between accuracy and robustness. Finally, we contaminated the dataset used in Fig 2C (sample size 3000) by randomly replacing one count per batch with the outlier value 30 and applied the same evaluation protocol. As shown in Fig 2H, the PGF-based inference method exhibits only a slight increase in inference error, whereas MOM shows a substantial degradation. This confirms that the PGF-based method is the most robust among the three.

In summary, the PGF-based inference method, when combined with the integration range [0,1], achieves the best overall performance in terms of accuracy, robustness, and computational efficiency (second only to MOM in speed).

### Extension to time-resolved count data

**Fig 3:**
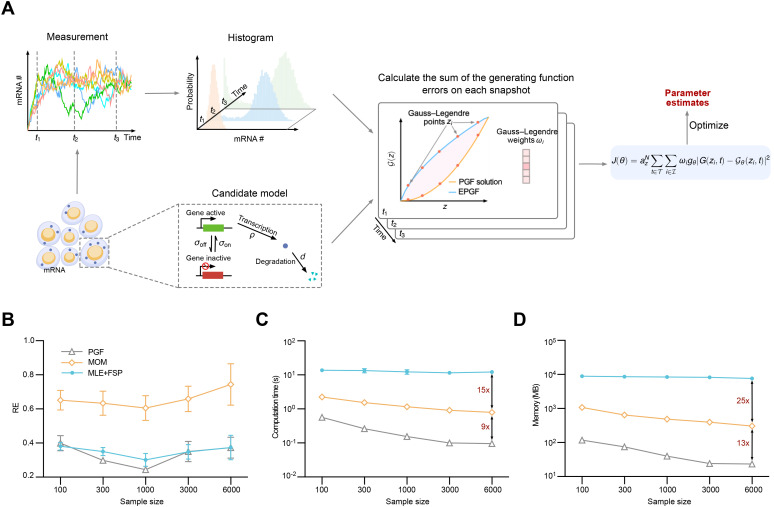
Performance of inference methods on time-resolved count data. A: Schematic of the PGF-based inference framework applied to time-resolved data. B: Inference accuracy across varying numbers of cells per snapshot (*n*_*c*_) and time points **(*n***_***t***_), with the total number of cells fixed at *n*_*c*_ × *n*_*t*_ = 12000. All methods exhibit an optimal trade-off near *n*_*c*_ = 1000 and *n*_*t*_ = 12, with the PGF-based method consistently achieving the highest accuracy. C: Computational time usage as a function of *n*_*c*_. D: Memory usage as a function of *n*_*c*_. The PGF-based method is the most efficient, outperforming the other two by one to two orders of magnitude.

Techniques such as single-molecule fluorescent in situ hybridization (smFISH), live-cell imaging, and single-cell EU RNA sequencing (scEU-seq) provide rich time-resolved count data for gene expression dynamics [11,47–49]. This motivates an extension of our PGF-based inference method to accommodate time-resolved data. Fortunately, this extension is straightforward to implement. The framework is illustrated in Fig 3A, using the telegraph model as a representative example. We assume that population-level snapshots of mRNA counts are collected at a set of discrete time points $𝒯=\{t_{1},t_{2},\ldots ,t_{n_{t}}\}$. For each time point t∈𝒯, we compute the EPGF *G*(*z*, *t*). In parallel, we evaluate the corresponding analytical PGF solution ${𝒢}_{\theta }(z,t)$ from the model at each time poin*t*. The discrepancy between the empirical and analytical PGFs is computed analogously to Eq. (8), leading to the following objective function


$$J(\theta )=a_{z}^{N}\sum_{t\in 𝒯}\sum_{𝐢\in ℐ}\omega_{𝐢}g_{\theta }|G(𝐳,t)-{𝒢}_{\theta }(𝐳,t){|}^{2}.$$ (9)

By substituting Eq. (9) for Eq. (8) in Algorithm 1, we obtain a natural extension of the PGF-based inference method for time-resolved count data.

Next, we compared the three inference methods using time-resolved count data. To do so, we reused the kinetic parameters from Fig 2C and supplemented them with a degradation rate of *d* = 1. Starting from the initial condition of an active gene with no mRNA present, we used SSA to simulate trajectories over the interval t∈[0,6]. We varied the number of snapshots (*n*_*t*_), evenly spaced over (0,6], from 120 to 2, and correspondingly varied the number of cells per snapshot (*n*_*c*_) from 100 to 6000, while keeping the total number of cells fixed at *n*_*c*_ × *n*_*t*_ = 12000. We then followed the same evaluation protocol used in Fig 2C to compare the three inference methods. Technical details for MOM and MLE + FSP are provided in the MOM-based inference method and MLE-based inference method section, respectively. To ensure consistency, the optimization hyperparameters were set to g_tol = 10^−10^, f_reltol = 10^−8^, and iterations = 2000. As shown in Fig 3B, all three methods exhibit a clear trade-off between temporal resolution (*n*_*t*_) and the number of cells per snapshot (*n*_*c*_), with the best performance occurring around *n*_*c*_ = 1000 and *n*_*t*_ = 12. This indicates that, under a fixed total sampling budget (*n*_*c*_ × *n*_*t*_), over-allocating the budget to temporal resolution (i.e., using many time points) reduces the number of cells per snapshot, increases snapshot-level uncertainty, and ultimately degrades parameter-estimation accuracy. Conversely, over-allocating the budget to the number of cells per snapshot reduces snapshot uncertainty but yields sparse temporal sampling, which is insufficient to resolve the dynamics accurately. Therefore, an optimal balance exists between these two extremes. Across the entire range of *n*_*c*_, the PGF-based method consistently achieved the highest accuracy. Interestingly, we also quantified the computational time (Fig 3C) and memory usage (Fig 3D) for all three methods. In this setting, the PGF-based method emerged as the most computationally efficient—it was an order of magnitude faster than MOM and used only one-tenth of its memory. This improvement arises because, unlike in the steady-state setting where MOM solves only algebraic equations, the time-resolved setting requires MOM to repeatedly solve ODEs for moment trajectories—an overhead that the PGF-based method avoids.

### Model selection using PGF-based inference for time-resolved count data

We now describe how to extend the PGF-based inference method for time-resolved count data to address the problem of model selection, with the goal of identifying gene activity dynamics. Since our method does not rely on conventional likelihood functions, classical model selection approaches based on information criteria (e.g., AIC [50], BIC [51]) are not applicable. Instead, we adopt and extend the cross-validation-based strategy proposed in Ref. [32], which was originally developed for steady-state count data.

Assume we collect count data from *n*_*c*_ cells at each time point t∈𝒯, where $𝒯=\{t_{1},t_{2},\ldots ,t_{n_{t}}\}$. To implement 10-fold cross-validation, we randomly partition the *n*_*c*_ cell-level observations at each time point into 10 equally sized subsamples. For each candidate model, nine subsamples are used to infer the kinetic parameters $\hat{\theta }$, and the remaining subsample serves as validation data, on which the inference accuracy is evaluated via the performance score $J(\hat{\theta })$ computed from Eq. (9). This process is repeated ten times so that each subsample is used exactly once for validation. The resulting vector of performance scores for each candidate model is denoted by ${𝒥}_{\text{model}}=[J({\hat{\theta }}_{1}),\ldots ,J({\hat{\theta }}_{10}){]}^{⊤}.$ To determine the best-fitting model, we apply the one-standard-error rule [52]. Given a set of competing models $\left\{{\text{model}}_{1},\ldots ,{\text{model}}_{n}\right\}$, we compute the mean and standard deviation of performance scores for each model


$${\bar{𝒥}}_{{\text{model}}_{i}}=\langle {𝒥}_{{\text{model}}_{i}}\rangle ,\;\sigma_{{\text{model}}_{i}}=\sigma ({𝒥}_{{\text{model}}_{i}})$$

for i=1,⋯,n. We identify the model with the lowest mean performance score,


$${\bar{𝒥}}_{\text{best}}=\min_{i=1,\ldots ,n}{\bar{𝒥}}_{{\text{model}}_{i}},$$

and denote its corresponding standard deviation as $\sigma_{\text{best}}$. We then compute the Pearson correlation coefficient between the performance score vector of the best model and that of each candidate model:


$$\rho_{\text{best},i}=\text{cor}({𝒥}_{\text{best}},{𝒥}_{{\text{model}}_{i}}).$$

The model-specific performance threshold is defined as


$${\text{thresh}}_{{\text{model}}_{i}}={\bar{𝒥}}_{\text{best}}+\sigma_{\text{best}}\sqrt{1-\rho_{\text{best},i}}.$$ (10)

**Fig 4:**
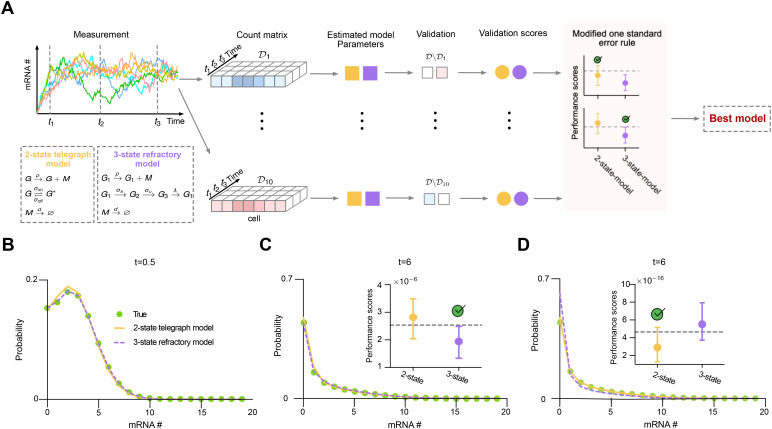
Validation of the PGF-based model selection method using time-resolved count data. A: Schematic of the PGF-based model selection framework applied to time-resolved data, using the telegraph and refractory models as candidate models (inset). B and C: Reconstructed mRNA distributions at *t* = 0.5 and *t* = 6 using inferred parameters from the refractory model (yellow) and the telegraph model (purple) based on one fold of time-resolved data. The refractory model matches the ground truth distribution (green) more closely than the telegraph model. (C, inset) Performance scores across 10 folds identify that the refractory model is correctly selected as the best-fitting model. D: Using only steady-state data at *t* = 6 results in incorrect selection of the telegraph model (inset). The reconstructed distribution based on the refractory model under this setting fails to capture the ground-truth distribution, particularly at zero-mRNA count.

A candidate model is considered competitive if its mean performance score is below this threshold. The full procedure is illustrated in Fig 4A and detailed in Model selection using PGF-based inference method section.

To validate the proposed model selection method, we considered the refractory model [42,53], a three-state gene model in which two states are transcriptionally inactive (prohibitive), and the remaining state permits active transcription, as illustrated in the inset of Fig 4A. Using the kinetic parameters reported in Table C in S1 Text, we employed the SSA to simulate 1,000 cells from time *t* = 0 to *t* = 6, starting from gene state *G*_1_ and zero mRNA. By *t* = 6, the system reaches steady state. Count data were collected at 0.5 time unit intervals. We evaluated model selection performance by listing the two-state telegraph model as a competing alternative and deriving the time-dependent PGF solution for the refractory model analytically (Section B in S1 Text). Applying the cross-validation–based PGF inference procedure to the time-resolved dataset, the resulting performance scores (Fig 4C, inset) correctly identified the refractory model as the best-fitting one. This conclusion is further supported by the reconstructed distributions: as shown in Fig 4B and 4C, the distributions reconstructed from inferred parameters using both the refractory and telegraph models (based on a representative fold; see Table C in S1 Text) were compared with the ground-truth distribution. The refractory model yields an more accurate match.

For comparison, we also applied the steady-state model selection method from Ref. [32], using only the snapshot at *t* = 6. In this case, the method incorrectly identified the telegraph model as the best-fitting one (Fig 4D, inset). The reconstructed distribution from the refractory model under this steady-state-only setting poorly captures the ground-truth, particularly at zero-mRNA levels, suggesting possible overfitting in parameter inference across folds. This is also reflected in the inferred parameter values (Table C in S1 Text), where estimates based on steady-state data are considerably less accurate than those obtained using time-resolved data—a trend also noted in Ref. [32]. Taken together, these results demonstrate the effectiveness of the PGF-based inference framework combined with cross-validation for model selection using time-resolved count data. Moreover, they highlight the necessity of time-resolved measurements for accurately identifying gene regulatory mechanisms.

## Discussion

In this paper, we extended the PGF-based inference method proposed in Ref. [32]—originally developed for steady-state count data—to accommodate time-resolved count data, and further generalized the associated model selection strategy based on cross-validation. Using this extended framework, we demonstrate that time-resolved data enables the reliable identification of complex gene expression models with multiple gene states, a task that cannot be achieved using traditional steady-state count data alone. In addition, we investigated the effect of key hyperparameters on inference accuracy and identified an optimal configuration for practical use. We systematically evaluated the accuracy, computational efficiency, and robustness under two types of data contamination, of representative methods from four major inference frameworks. Our results show that the PGF-based inference method consistently outperforms the others across nearly all experimental settings and evaluation metrics. These findings highlight the PGF-based approach as a highly promising next-generation inference framework for count data, a common data structure arising in stochastic biochemical reaction systems.

PGF-based inference methods have also been studied in Refs. [30,31], where inference is performed by minimizing the density power divergence, which involves a hyperparameter α. In this work, we use the simpler, numerically more stable mean squared error metric, consistent with Ref. [32] and with the approach adopted throughout this paper. It is worth noting that Refs. [30,31] primarily focused on models with simple analytical PGFs, such as the Poisson and negative binomial distributions. In contrast, the PGFs addressed in Ref. [32] and in the present work arise from biochemical kinetic models and are substantially more complex.

One limitation of PGF-based inference is its dependence on analytical PGF solutions, which are generally unavailable for arbitrary reaction networks. However, this limitation can be partially alleviated in two ways: (i) the PGF solutions summarized in Table A of S1 Text can be extended to more complex networks using the properties listed in Section A of S1 Text; and (ii) newer approaches, such as the queueing-theoretic framework in Ref. [32], enable PGF-based solutions for broader classes of stochastic reaction networks. As analytical solutions continue to accumulate and more advanced solution techniques are developed, the computational efficiency and accuracy of PGF-based inference become increasingly valuable. In this context, a key contribution of PGF-based inference is that it bridges the rich theoretical literature on PGF solutions with practical analysis of scRNA-seq data.

The primary goal of this paper is to provide a systematic evaluation of the computational efficiency, accuracy, and robustness of the PGF-based inference method. In particular, assessing accuracy requires ground-truth parameter values, which are typically unavailable in experimental datasets but can be specified in synthetic data; accordingly, we rely extensively on synthetic datasets throughout this study. While applying the PGF-based inference method to large-scale scRNA-seq datasets could enable deeper biological analysis, such applications are beyond the scope of the present paper.

Notably, the PGF-based inference method proposed in Ref. [32] and further developed in the present paper is readily extensible to multi-species biochemical reaction systems. Although this study focuses on the telegraph model involving a single mRNA species - so as to isolate and evaluate inference performance without confounding factors - this extensibility is a key feature for developing kinetic models based on the central dogma of molecular biology. This is particularly important in light of recent advances in single-cell sequencing technologies, which allow for simultaneous measurement of multiple molecular species within the same cell. For example, cellular indexing of transcriptomes and epitopes by sequencing (CITE-seq) enables joint quantification of mRNA and surface proteins [54], single-cell assay for transposase-accessible chromatin using sequencing (scATAC-seq) captures chromatin accessibility alongside transcriptomic data [55], multiplexed error-robust fluorescence in situ hybridization (MERFISH) provides spatially resolved nuclear and cytoplasmic RNA counts [56], and Velocyto extracts spliced and unspliced RNA counts [57]. These developments highlight the importance of modeling frameworks that can flexibly incorporate multiple species.

While the present study focuses on the application of the PGF-based inference method to model selection, future work may explore its integration with other downstream tasks, such as clustering and deconvolution, to further leverage the power of PGF in single-cell data analysis.

## Materials and methods

### MOM-based inference method

One competing approach is the MOM-based inference method, which constructs a synthetic likelihood from the moments of the count data. For clarity, we focus here on the procedure for applying the MOM-based method to infer the kinetic parameters of the telegraph model.

Consider a population of *n*_*c*_ cells, where each cell has *n*_*i*_(*t*_*j*_) molecules of species *X* (e.g., mRNA) measured at time *t*_*j*_, for $j=1,2,\ldots ,n_{t}$. The first three moments compu*t*ed from the count data are


$$\mu_{k}(t_{j})=\{\begin{array}{ll} \frac{1}{n_{c}}\sum_{i=1}^{n_{c}}n_{i}(t_{j}) & k=1\\ \frac{1}{n_{c}}\sum_{i=1}^{n_{c}}(n_{i}(t_{j})-\mu_{1}(t_{j}){)}^{k} & k=2,3 \end{array}$$ (11)

By the law of large numbers, the moment distribution is approximately Gaussian. We use the following likelihood function [10,12] to infer the kinetic parameters


$$ℒ\left[\mu_{1}(t_{j}),\cdots ,\mu_{k}(t_{j})|\theta \right]=\prod_{j=1}^{n_{t}}\prod_{k=1}^{n_{k}}\frac{1}{\sqrt{2\pi \sigma_{k}^{2}(t_{j})}}\text{exp}\left(-\frac{{\left(\mu_{k}(t_{j})-{\hat{\mu }}_{k}(\theta ,t_{j})\right)}^{2}}{2\sigma_{k}^{2}(t_{j})}\right),$$ (12)

where $\sigma_{k}^{2}(t_{j})$ denotes the variance of the *k*-th order empirical moment at time *t*_*j*_, computed from the count da*t*a using the following expressions


$$\begin{array}{cc} & \sigma_{1}^{2}(t_{j})=\frac{1}{n_{c}}\mu_{2}^{2}(t_{j}),\\ & \sigma_{2}^{2}(t_{j})=\frac{1}{n_{c}}\left(\mu_{4}(t_{j})-\frac{n_{c}-3}{n_{c}-1}\mu_{2}^{2}(t_{j})\right),\\ & \sigma_{3}^{2}(t_{j})=\frac{1}{n_{c}}\left(\mu_{6}(t_{j})-\mu_{3}^{2}(t_{j})\right). \end{array}$$ (13)

By contrast, the moments ${\hat{\mu }}_{k}(\theta ,t_{j})$ are theoretical moments computed from the underlying kinetic model. For the telegraph model under steady-state conditions, the four kinetic parameters cannot be independently identified; only the ratios of ρ, $\sigma_{\text{on}}$, and $\sigma_{\text{off}}$ normalized by the degradation rate *d* are identifiable. Therefore, we fix *d* = 1 without loss of generality. In this setting, we set the number of moments *n*_*k*_ = 3 and the number of time points *n*_*t*_ = 1, so that ${\hat{\mu }}_{k}(\theta ,t_{j})$ simplifies to ${\hat{\mu }}_{k}(\theta )$. These moments can be directly derived from the steady-state PGF solution provided in Table A in S1 Text, and are given by


$$\begin{array}{cc} {\hat{\mu }}_{1}(\theta )= & \frac{\rho \sigma_{\text{on}}}{d(\sigma_{\text{off}}+\sigma_{\text{on}})},\\ {\hat{\mu }}_{2}(\theta )= & \frac{\rho \sigma_{\text{on}}}{d(\sigma_{\text{off}}+\sigma_{\text{on}})}+\frac{\rho^{2}\sigma_{\text{off}}\sigma_{\text{on}}}{d(\sigma_{\text{off}}+\sigma_{\text{on}}{)}^{2}(d+\sigma_{\text{off}}+\sigma_{\text{on}})},\\ {\hat{\mu }}_{3}(\theta )= & \frac{\rho \sigma_{\text{on}}}{d(\sigma_{\text{off}}+\sigma_{\text{on}})}+\frac{3\rho^{2}\sigma_{\text{off}}\sigma_{\text{on}}}{d(\sigma_{\text{off}}+\sigma_{\text{on}}{)}^{2}(d+\sigma_{\text{off}}+\sigma_{\text{on}})}\\ & +\frac{2\rho^{3}\sigma_{\text{off}}\sigma_{\text{on}}(\sigma_{\text{off}}-\sigma_{\text{on}})}{d(\sigma_{\text{off}}+\sigma_{\text{on}}{)}^{3}(d+\sigma_{\text{off}}+\sigma_{\text{on}})(2d+\sigma_{\text{off}}+\sigma_{\text{on}})}, \end{array}$$ (14)

with *d* = 1. Maximizing the likelihood defined in Eq. (12) is equivalent to minimizing its negative logarithmic likelihood, which is given by


$$J_{\text{MOM}}(\theta )=\sum_{k=1}^{3}\frac{{\left(\mu_{k}-{\hat{\mu }}_{k}(\theta )\right)}^{2}}{\sigma_{k}^{2}}.$$ (15)

Under steady-state conditions, the numerical procedure for the MOM-based inference method is outlined in Algorithm 2, with optimization details identical to those of the PGF-based inference method.

**Algorithm 2 MOM-based inference method**

**Input:** Number of cells (*n*_*c*_), the count vector $\left\{n_{1},\cdots ,n_{c}\right\}$

**Output:** Kinetic parameters θ.

1:  Initialize the inferred parameters θ

2:  Compute the moment $\mu_{k}$ and variance $\sigma_{k}^{2}$ from count vector using Eqs. (11) and Eq. (13)

3:  **while** Threshold not reached **do**

4:   Use Eq. (14) to compute the moments of the telegraph model

5:   Employ the Nelder-Mead optimization algorithm to solve Eq. (15) and update the inferred parameters θ

6:  **end while**

7:  **return** Kinetic parameters θ

Indeed, Algorithm 2 under the steady state conditions are employed as a parameter initialization strategy in Fig 1D.

To extend Algorithm 2 to time-resolved count data, we set the number of moments to *n*_*k*_ = 2. In this setting, the reduction in the number of moment measurements is compensated by increased temporal resolution across multiple time points. The number of kinetic parameters to be inferred is four. The theoretical moments ${\hat{\mu }}_{k}(\theta ,t_{j})$ at each time point *t*_*j*_ are computed by solving the system of momen*t* equations


$$\left\{ \begin{array}{c} \partial_{t}\langle n_{m}\rangle =\rho \langle n_{g}\rangle -d\langle n_{m}\rangle ,\\ \partial_{t}\langle n_{g}\rangle =-\sigma_{\text{off}}\langle n_{g}\rangle +\sigma_{\text{on}}\left(1-\langle n_{g}\rangle \right),\\ \partial_{t}\langle n_{m}^{2}\rangle =2\rho \langle n_{m}n_{g}\rangle +d\langle n_{m}\rangle -2d\langle n_{m}^{2}\rangle +\rho \langle n_{g}\rangle ,\\ \partial_{t}\langle n_{m}n_{g}\rangle =\rho \langle n_{g}\rangle +\sigma_{\text{on}}\langle n_{m}\rangle -(d+\sigma_{\text{off}}+\sigma_{\text{on}})\langle n_{m}n_{g}\rangle , \end{array}\right.$$ (16)

where ⟨·⟩ denotes the expected value. Solving this system yields $\langle n_{m}\rangle$ and $\langle n_{m}^{2}\rangle$ at each time point *t*_*j*_, for $j=1,\ldots ,n_{t}$. These are used to compute the first and second theoretical moments ${\hat{\mu }}_{1}(t_{j})$ and ${\hat{\mu }}_{2}(t_{j})$. Accordingly, for time-resolved count data, Algorithm 2 is modified as follows: (i) In S*t*ep 2, the empirical moments $\mu_{k}(t_{j})$ are computed for *k* = 1, 2 across all time points. (ii) In Step 4, the theoretical moments ${\hat{\mu }}_{k}(\theta ,t_{j})$ are obtained by numerically solving the moment equations in Eq. (16). (iii) In Step 5, the loss function defined in Eq. (12) becomes


$$J_{\text{MOM}}(\theta )=\sum_{j=1}^{n_{t}}\sum_{k=1}^{2}\frac{{\left(\mu_{k}(t_{j})-{\hat{\mu }}_{k}(\theta ,t_{j})\right)}^{2}}{\sigma_{k}^{2}(t_{j})}.$$

### MLE-based inference method

As MLE-based methods are commonly used and serve as natural benchmarks for comparison, we provide the technical details of the MLE-based approach that utilizes the FSP method for likelihood computation.

Given observations from *n*_*c*_ cells measured at time points *t*_*j*_ for $j=1,\ldots ,n_{t}$, the dataset for *N* molecular species is denoted as $𝒟=\{(n_{i1}(t_{j}),\cdots ,n_{iN}(t_{j}))\}$, where *n*_*ik*_(*t*_*j*_) is the copy number of species *k* in cell *i* at time *t*_*j*_. The total likelihood of observing all data is given by the product over all cells and time points


$$ℒ(𝒟|\theta )=\prod_{j=1}^{n_{t}}\prod_{i=1}^{n_{c}}P(n_{i1}(t_{j}),\cdots ,n_{iN}(t_{j})|\theta ).$$

Inference of the kinetic parameters θ is then performed by minimizing the negative log-likelihood


$$J_{\text{MLE}}(\theta )=-\sum_{j=1}^{n_{t}}\sum_{i=1}^{n_{c}}\log P\left(n_{i1}(t_{j}),\ldots ,n_{iN}(t_{j})|\theta \right).$$ (17)

The probability $P(n_{i1}(t_{j}),\cdots ,n_{iN}(t_{j})|\theta )$ is computed using FSP, which approximates the solution of CMEs by solving a truncated system of ODEs [19]. Specifically, the truncated CME for the telegraph model is given by


$$\frac{\text{d}𝐏(t|\theta )}{\text{d}t}=𝐀𝐏(t|\theta ),$$ (18)

where the probability vector is defined as


$$𝐏(t)=[P_{0}(0,t|\theta ),\cdots ,P_{0}(n_{T},t|\theta ),P_{1}(0,t|\theta ),\cdots ,P_{1}(n_{T},t|\theta ){]}^{⊤},$$ (19)

with $P_{s}(n,t|\theta )$ denoting the probability of observing *n* mRNA molecules while the gene is in state s∈{0,1} at time *t*, and *n*_*T*_ representing the state space truncation level. The transi*t*ion rate matrix **A** has the block structure,


$$\begin{array}{c} 𝐀=\left[\begin{array}{cc} {𝐀}_{1} & {𝐀}_{2}\\ {𝐀}_{3} & {𝐀}_{4} \end{array}\right]. \end{array}$$ (20)

Here the submatrices are given by


$$\begin{array}{cc} {𝐀}_{1}= & -\sigma_{\text{on}}𝐈-\text{diag}([0,d,\cdots ,n_{T}d])+{\text{diag}}_{1}([d,\cdots ,n_{T}d]),\\ {𝐀}_{2}= & \sigma_{\text{off}}𝐈,\\ {𝐀}_{3}= & \sigma_{\text{on}}𝐈,\\ {𝐀}_{4}= & -(\sigma_{\text{off}}+\rho )𝐈-\text{diag}([0,d,\cdots ,n_{T}d])+{\text{diag}}_{1}([d,\cdots ,n_{T}d])\\ & +{\text{diag}}_{-1}(\rho 𝐈). \end{array}$$ (21)

The operator ${\text{diag}}_{\varphi }(𝐯)$ constructs a diagonal matrix with the elements of the vector **v** placed on the main diagonal when there is no subscript, on the upper off-diagonal when φ=1, and on the lower off-diagonal when φ=−1. The identity matrix is denoted as **I**. This system is numerically integrated using standard ODE solvers to evaluate the likelihood required for MLE. Notably, CMEs of any kinetic model can be concisely expressed in the form of Eq. (18) by organizing the probabilities of all possible states into the vector 𝐏(t|θ).

The numerical procedure for the MLE-based inference method is outlined in Algorithm 3, with optimization details identical to those of the PGF-based inference method.

**Algorithm 3 MLE-based inference method**

**Input:** Number of cells (*n*_*c*_), number of snapshots in time (*n*_*t*_), the count tuples of *N* species $\left\{(n_{i1}(t_{j}),\cdots ,n_{iN}(t_{j}))\right\}$ for $i=1,\cdots ,n_{c}$ and $j=1,\cdots ,n_{t}$

**Output:** Kinetic parameters θ.

1:  Initialize the inferred parameters θ

2:  **while** Threshold not reached **do**

3:   Compute the probability $P(n_{i1}(t_{j}),\cdots ,n_{iN}(t_{j}))|\theta )$ for the inferred parameters θ using Eq. (18)

4:   Employ the Nelder-Mead optimization algorithm to solve Eq. (17) and update the inferred parameters θ

5:  **end while**

6:  **return** Kinetic parameters θ

It should be noted that under steady-state conditions (i.e., *n*_*t*_ = 1), there is no need to integrate Eq. (18) over time to obtain the steady-state distribution. Instead, one can directly solve the corresponding stationary system by modifying the equation as follows: replace the first row of the matrix **A** with all ones, and set the left-hand side of Eq. (18) to the vector $[1,0,\ldots ,0{]}^{⊤}$. Solving this modified set of algebraic equations yields the steady-state probability $P(n_{i1},\ldots ,n_{iN}|\theta )$, which is used in Step 3 of Algorithm 3.

### Model selection using PGF-based inference method

**Algorithm 4 Model selection method**

**Input:** Number of cells (*n*_*c*_), the equal sized counts data $\left\{{𝒟}_{1},{𝒟}_{2},\cdots ,{𝒟}_{10}\right\}$, the set of candidate models $\left\{{\text{model}}_{1},\cdots ,{\text{model}}_{n}\right\}$ ordered by the model complexity (the number of kinetic parameters)

**Output:** Best-fitting model

1:  **for** each candidate model model_*i*_ **do**

2:   **for** each fold *j* **do**

3:    Use Algorithm 1 to infer kinetic parameters ${\hat{\phi }}_{j}$ based on the data $\left\{{𝒟}_{1},\cdots ,{𝒟}_{j},{𝒟}_{j+1},\cdots ,{𝒟}_{10}\right\}$

4:    Compute the performance score $J({\hat{\phi }}_{j})$ on the validation dataset ${𝒟}_{j}$

5:   **end for**

6:   Collect all the performance scores for model_*i*_ ${𝒥}_{{\text{model}}_{i}}=[J({\hat{\phi }}_{1}),\cdots ,J({\hat{\phi }}_{10}){]}^{⊤}$

7:   Compute the corresponding mean (${\bar{𝒥}}_{{\text{model}}_{i}}$) and standard deviation $\sigma_{{\text{model}}_{i}}$

8:  **end for**

9:  Find the minimal performance score ${\bar{𝒥}}_{\text{best}}$ and its index ℐ

10:  **for** each candidate model model_*i*_ and i≤ℐ **do**

11:   Calculate the correlation coefficient $\rho_{\text{best},i}$ of the performance score vectors of the best model and model_*i*_

12:   Calculate the threshold of performance score ${\text{thresh}}_{{\text{model}}_{i}}$ using Eq. (10)

13:   **if** ${\bar{𝒥}}_{{\text{model}}_{i}}\leq {\text{thresh}}_{{\text{model}}_{i}}$ **then**

14:    Accept model_*i*_ as the best-fitting model

15:    Break

16:   **end if**

17:  **end for**

18:  **return** Best-fitting model model_*i*_

## Supporting information

S1 Text Supplemental Notes, Supplemental Tables, and References. This appendix includes a summary table of exact probability generating function (PGF) solutions for a broad class of stochastic gene-expression models, including birth–death, bursty, telegraph, refractory, feedback, delayed-degradation, and two-compartment extensions (Table A). It also presents the key properties of PGFs used throughout this work, including binomial partitioning, marginalization, summation, independence, and zero inflation (Section A). In addition, the appendix provides a detailed derivation of the exact time-dependent solution for the three-state refractory model (Section B). References are listed at the end of the appendix.
(PDF)

## References

[1] Elowitz MB, Levine AJ, Siggia ED, Swain PS. Stochastic gene expression in a single cell. Science. 2002;297(5584):1183–6. doi: 10.1126/science.1070919 12183631

[2] Blake WJ, KAErn M, Cantor CR, Collins JJ. Noise in eukaryotic gene expression. Nature. 2003;422(6932):633–7. doi: 10.1038/nature01546 12687005

[3] Rodriguez J, Ren G, Day CR, Zhao K, Chow CC, Larson DR. Intrinsic Dynamics of a Human Gene Reveal the Basis of Expression Heterogeneity. Cell. 2019;176(1–2):213-226.e18. doi: 10.1016/j.cell.2018.11.026 30554876 PMC6331006

[4] Sanchez A, Golding I. Genetic determinants and cellular constraints in noisy gene expression. Science. 2013;342(6163):1188–93. doi: 10.1126/science.1242975 24311680 PMC4045091

[5] Raser JM, O’Shea EK. Control of stochasticity in eukaryotic gene expression. Science. 2004;304(5678):1811–4. doi: 10.1126/science.1098641 15166317 PMC1410811

[6] Karr JR, Sanghvi JC, Macklin DN, Gutschow MV, Jacobs JM, Bolival B Jr, et al. A whole-cell computational model predicts phenotype from genotype. Cell. 2012;150(2):389–401. doi: 10.1016/j.cell.2012.05.044 22817898 PMC3413483

[7] Thornburg ZR, Bianchi DM, Brier TA, Gilbert BR, Earnest EE, Melo MCR, et al. Fundamental behaviors emerge from simulations of a living minimal cell. Cell. 2022;185(2):345-360.e28. doi: 10.1016/j.cell.2021.12.025 35063075 PMC9985924

[8] Van Kampen NG. Stochastic processes in physics and chemistry. vol. 1. Elsevier. 1992.

[9] Gardiner CW. Handbook of stochastic methods. vol. 3. Berlin: Springer; 2004.

[10] Cao Z, Grima R. Accuracy of parameter estimation for auto-regulatory transcriptional feedback loops from noisy data. J R Soc Interface. 2019;16(153):20180967. doi: 10.1098/rsif.2018.0967 30940028 PMC6505555

[11] Neuert G, Munsky B, Tan RZ, Teytelman L, Khammash M, van Oudenaarden A. Systematic identification of signal-activated stochastic gene regulation. Science. 2013;339(6119):584–7. doi: 10.1126/science.1231456 23372015 PMC3751578

[12] Zechner C, Ruess J, Krenn P, Pelet S, Peter M, Lygeros J, et al. Moment-based inference predicts bimodality in transient gene expression. Proc Natl Acad Sci U S A. 2012;109(21):8340–5. doi: 10.1073/pnas.1200161109 22566653 PMC3361437

[13] Ljung L. System identification. In: Signal analysis and prediction. Springer; 1998. p. 163–173.

[14] Ljung L. Perspectives on system identification. Ann Rev Cont. 2010;34(1):1–12. doi: 10.1016/j.arcontrol.2009.12.001

[15] Fu X, Patel HP, Coppola S, Xu L, Cao Z, Lenstra TL, et al. Quantifying how post-transcriptional noise and gene copy number variation bias transcriptional parameter inference from mRNA distributions. Elife. 2022;11:e82493. doi: 10.7554/eLife.82493 36250630 PMC9648968

[16] Munsky B, Li G, Fox ZR, Shepherd DP, Neuert G. Distribution shapes govern the discovery of predictive models for gene regulation. Proc Natl Acad Sci U S A. 2018;115(29):7533–8. doi: 10.1073/pnas.1804060115 29959206 PMC6055173

[17] Skinner SO, Xu H, Nagarkar-Jaiswal S, Freire PR, Zwaka TP, Golding I. Single-cell analysis of transcription kinetics across the cell cycle. Elife. 2016;5:e12175. doi: 10.7554/eLife.12175 26824388 PMC4801054

[18] Schnoerr D, Sanguinetti G, Grima R. Approximation and inference methods for stochastic biochemical kinetics—a tutorial review. J Phys A: Math Theor. 2017;50(9):093001. doi: 10.1088/1751-8121/aa54d9

[19] Munsky B, Khammash M. The finite state projection algorithm for the solution of the chemical master equation. J Chem Phys. 2006;124(4):044104. doi: 10.1063/1.2145882 16460146

[20] Munsky B, Khammash M. The Finite State Projection Approach for the Analysis of Stochastic Noise in Gene Networks. IEEE Trans Automat Contr. 2008;53(Special Issue):201–14. doi: 10.1109/tac.2007.911361

[21] Milner P, Gillespie CS, Wilkinson DJ. Moment closure based parameter inference of stochastic kinetic models. Stat Comput. 2012;23(2):287–95. doi: 10.1007/s11222-011-9310-8

[22] Komorowski M, Finkenstädt B, Harper CV, Rand DA. Bayesian inference of biochemical kinetic parameters using the linear noise approximation. BMC Bioinformatics. 2009;10:343. doi: 10.1186/1471-2105-10-343 19840370 PMC2774326

[23] Stathopoulos V, Girolami MA. Markov chain Monte Carlo inference for Markov jump processes via the linear noise approximation. Philos Trans A Math Phys Eng Sci. 2012;371(1984):20110541. doi: 10.1098/rsta.2011.0541 23277599

[24] Fearnhead P, Giagos V, Sherlock C. Inference for reaction networks using the linear noise approximation. Biometrics. 2014;70(2):457–66. doi: 10.1111/biom.12152 24467590

[25] Cao Z, Grima R. Linear mapping approximation of gene regulatory networks with stochastic dynamics. Nat Commun. 2018;9(1):3305. doi: 10.1038/s41467-018-05822-0 30120244 PMC6098115

[26] Singh A, Hespanha JP. A derivative matching approach to moment closure for the stochastic logistic model. Bull Math Biol. 2007;69(6):1909–25. doi: 10.1007/s11538-007-9198-9 17443391

[27] Wu Q, Smith-Miles K, Tian T. Approximate Bayesian computation schemes for parameter inference of discrete stochastic models using simulated likelihood density. BMC Bioinformatics. 2014;15(Suppl 12):S3. doi: 10.1186/1471-2105-15-S12-S3 25473744 PMC4243104

[28] Toni T, Welch D, Strelkowa N, Ipsen A, Stumpf MPH. Approximate Bayesian computation scheme for parameter inference and model selection in dynamical systems. J R Soc Interface. 2009;6(31):187–202. doi: 10.1098/rsif.2008.0172 19205079 PMC2658655

[29] Loos C, Marr C, Theis FJ, Hasenauer J. Approximate Bayesian Computation for stochastic single-cell time-lapse data using multivariate test statistics. In: International Conference on Computational Methods in Systems Biology. Springer; 2015. p. 52–63.

[30] Basu A. Robust and efficient estimation by minimising a density power divergence. Biometrika. 1998;85(3):549–59. doi: 10.1093/biomet/85.3.549

[31] Tay SY, Ng CM, Ong SH. Parameter estimation by minimizing a probability generating function-based power divergence. Commun Stat Simulat Comput. 2018;48(10):2898–912. doi: 10.1080/03610918.2018.1468462

[32] Wang Y, Szavits-Nossan J, Cao Z, Grima R. Joint Distribution of Nuclear and Cytoplasmic mRNA Levels in Stochastic Models of Gene Expression: Analytical Results and Parameter Inference. Phys Rev Lett. 2025;135(6):068401. doi: 10.1103/q5sd-tpms 40864937

[33] Chari T, Gorin G, Pachter L. Biophysically interpretable inference of cell types from multimodal sequencing data. Nat Comput Sci. 2024;4(9):677–89. doi: 10.1038/s43588-024-00689-2 39317762

[34] Gorin G, Vastola JJ, Pachter L. Studying stochastic systems biology of the cell with single-cell genomics data. Cell Syst. 2023;14(10):822–43.e22. doi: 10.1016/j.cels.2023.08.004 37751736 PMC10725240

[35] Raj A, Peskin CS, Tranchina D, Vargas DY, Tyagi S. Stochastic mRNA synthesis in mammalian cells. PLoS Biol. 2006;4(10):e309. 10.1371/journal.pbio.0040309 PMC1563489 17048983

[36] Iyer-Biswas S, Hayot F, Jayaprakash C. Stochasticity of gene products from transcriptional pulsing. Phys Rev E Stat Nonlin Soft Matter Phys. 2009;79(3 Pt 1):031911. doi: 10.1103/PhysRevE.79.031911 19391975

[37] Grima R, Schmidt DR, Newman TJ. Steady-state fluctuations of a genetic feedback loop: an exact solution. J Chem Phys. 2012;137(3):035104. doi: 10.1063/1.4736721 22830733

[38] Cao Z, Grima R. Analytical distributions for detailed models of stochastic gene expression in eukaryotic cells. Proc Natl Acad Sci U S A. 2020;117(9):4682–92. doi: 10.1073/pnas.1910888117 32071224 PMC7060679

[39] Kumar N, Platini T, Kulkarni RV. Exact distributions for stochastic gene expression models with bursting and feedback. Phys Rev Lett. 2014;113(26):268105. doi: 10.1103/PhysRevLett.113.268105 25615392

[40] Wang Y, Yu Z, Grima R, Cao Z. Exact solution of a three-stage model of stochastic gene expression including cell-cycle dynamics. J Chem Phys. 2023;159(22):224102. doi: 10.1063/5.0173742 38063222

[41] Jiang Q, Fu X, Yan S, Li R, Du W, Cao Z, et al. Neural network aided approximation and parameter inference of non-Markovian models of gene expression. Nat Commun. 2021;12(1):2618. doi: 10.1038/s41467-021-22919-1 33976195 PMC8113478

[42] Cao Z, Filatova T, Oyarzún DA, Grima R. A Stochastic Model of Gene Expression with Polymerase Recruitment and Pause Release. Biophys J. 2020;119(5):1002–14. doi: 10.1016/j.bpj.2020.07.020 32814062 PMC7474183

[43] Jia C, Grima R. Holimap: an accurate and efficient method for solving stochastic gene network dynamics. Nat Commun. 2024;15(1):6557. doi: 10.1038/s41467-024-50716-z 39095346 PMC11297302

[44] Peccoud J, Ycart B. Markovian Modeling of Gene-Product Synthesis. Theor Popul Biol. 1995;48(2):222–34. doi: 10.1006/tpbi.1995.1027

[45] Fu X, Zhou X, Gu D, Cao Z, Grima R. DelaySSAToolkit.jl: stochastic simulation of reaction systems with time delays in Julia. Bioinformatics. 2022;38(17):4243–5. doi: 10.1093/bioinformatics/btac472 35799359

[46] Tang W, Bertaux F, Thomas P, Stefanelli C, Saint M, Marguerat S, et al. bayNorm: Bayesian gene expression recovery, imputation and normalization for single-cell RNA-sequencing data. Bioinformatics. 2020;36(4):1174–81. doi: 10.1093/bioinformatics/btz726 31584606 PMC7703772

[47] Donovan BT, Huynh A, Ball DA, Patel HP, Poirier MG, Larson DR, et al. Live-cell imaging reveals the interplay between transcription factors, nucleosomes, and bursting. EMBO J. 2019;38(12):e100809. doi: 10.15252/embj.2018100809 31101674 PMC6576174

[48] Volteras D, Shahrezaei V, Thomas P. Global transcription regulation revealed from dynamical correlations in time-resolved single-cell RNA sequencing. Cell Syst. 2024;15(8):694-708.e12. doi: 10.1016/j.cels.2024.07.002 39121860

[49] Battich N, Beumer J, de Barbanson B, Krenning L, Baron CS, Tanenbaum ME, et al. Sequencing metabolically labeled transcripts in single cells reveals mRNA turnover strategies. Science. 2020;367(6482):1151–6. doi: 10.1126/science.aax3072 32139547

[50] Akaike H. Factor Analysis and AIC. Psychometrika. 1987;52(3):317–32. doi: 10.1007/bf02294359

[51] Burnham KP, Anderson DR. Multimodel inference: understanding AIC and BIC in model selection. Sociol Methods Res. 2004;33(2):261–304.

[52] Yates LA, Aandahl Z, Richards SA, Brook BW. Cross validation for model selection: A review with examples from ecology. Ecol Monogr. 2023;93(1). doi: 10.1002/ecm.1557

[53] Suter DM, Molina N, Gatfield D, Schneider K, Schibler U, Naef F. Mammalian genes are transcribed with widely different bursting kinetics. Science. 2011;332(6028):472–4. doi: 10.1126/science.1198817 21415320

[54] Stoeckius M, Hafemeister C, Stephenson W, Houck-Loomis B, Chattopadhyay PK, Swerdlow H, et al. Simultaneous epitope and transcriptome measurement in single cells. Nat Methods. 2017;14(9):865–8. doi: 10.1038/nmeth.4380 28759029 PMC5669064

[55] Ranzoni AM, Tangherloni A, Berest I, Riva SG, Myers B, Strzelecka PM, et al. Integrative Single-Cell RNA-Seq and ATAC-Seq Analysis of Human Developmental Hematopoiesis. Cell Stem Cell. 2021;28(3):472-487.e7. doi: 10.1016/j.stem.2020.11.015 33352111 PMC7939551

[56] Chen KH, Boettiger AN, Moffitt JR, Wang S, Zhuang X. RNA imaging. Spatially resolved, highly multiplexed RNA profiling in single cells. Science. 2015;348(6233):aaa6090. doi: 10.1126/science.aaa6090 25858977 PMC4662681

[57] La Manno G, Soldatov R, Zeisel A, Braun E, Hochgerner H, Petukhov V, et al. RNA velocity of single cells. Nature. 2018;560(7719):494–8. doi: 10.1038/s41586-018-0414-6 30089906 PMC6130801

