# Supplementary material for: Efficiency, accuracy and robustness of probability generating function based parameter inference method for stochastic biochemical reactions
Source: PLoS Comput Biol. 2026 Apr 10;22(4):e1014160. doi: 10.1371/journal.pcbi.1014160 (PMC13068235; doi:10.1371/journal.pcbi.1014160)
Supplement: S1 Text — This appendix includes a summary table of exact probability generating function (PGF) solutions for a broad class of stochastic gene-expression models, including birth–death, bursty, telegraph, refractory, feedback, delayed-degradation, and two-compartment extensions (Table A). It also presents the key properties of PGFs used throughout this work, including binomial partitioning, marginalization, summation, independence, and zero inflation (Section A). In addition, the appendix provides a detailed derivation of the exact time-dependent solution for the three-state refractory model (Section B). References are listed at the end of the appendix. (PDF) [file pcbi.1014160.s001.pdf]

Supplemental Material

Contents

A. Properties of probability generating functions 5
B. Exact time-dependent solution of the refractory model 6

References 13

TABLE A. Zoo of PGF solutions: TD stands for time-dependent, SS stands for steady-state, BD stands for birth-death, Tele stands for telegraph, PRPR stands for polymerase recruitment and pause release, and DD stands for delayed degradation. $f(b,i) = b^i/(1+b)^{i+1}$, $u_{1,2} = z_{1,2} - 1$, $x_{1,2} = \rho u_{1,2}/d$, $M(.)$ stands for kummer confluent hypergeometric function.

**Model name**	**Reactions**	**TD/SS**	**PGF solution**	**Reference**
BD	$G \xrightarrow{\rho} G + M,\ M \xrightarrow{d} \varnothing$	SS	$\mathcal{G}(z_1) = \mathrm{e}^{x_1}$	/
		TD	$\mathcal{G}(z_1,t) = g(u_1\mathrm{e}^{-dt}) \exp\left[x_1(1-\mathrm{e}^{-dt})\right]$	
Bursty	$G \xrightarrow{\rho f(b,i)} G + iM,\ M \xrightarrow{d} \varnothing$	SS	$\mathcal{G}(z_1) = (1 - bu_1)^{-\frac{\rho}{d}}$	[1]
		TD	$\mathcal{G}(z_1,t) = g_1(u_1\mathrm{e}^{-dt}) \left(\frac{1-bu_1\mathrm{e}^{-dt}}{1-bu_1}\right)^{\frac{\rho}{d}}$	
Tele	$G \underset{\sigma_{\mathrm{on}}}{\overset{\sigma_{\mathrm{off}}}{\rightleftharpoons}} G^*,\ G \xrightarrow{\rho} G + M,\ M \xrightarrow{d} \varnothing$	SS	$\mathcal{G}(z_1) = M\left(\frac{\sigma_{\mathrm{on}}}{d}; \frac{\sigma_{\mathrm{off}}+\sigma_{\mathrm{on}}}{d}; x_1\right)$	[2]
		TD	$\mathcal{G}(z_1,t) = \begin{bmatrix}1 & 1\end{bmatrix} \begin{bmatrix}A_1 & A_2\\ A_3 & A_4\end{bmatrix} \begin{bmatrix}B_1 & B_2\\ B_3 & B_4\end{bmatrix} \begin{bmatrix}g_0(u_1\mathrm{e}^{-dt})\\ g_1(u_1\mathrm{e}^{-dt})\end{bmatrix},$<br>$A_1 = -\frac{\mathrm{e}^{x_1\exp(-dt)}}{\sigma_{\mathrm{off}}+\sigma_{\mathrm{on}}} M\left(1-\frac{\sigma_{\mathrm{off}}}{d}; 1-\frac{\sigma_{\mathrm{off}}+\sigma_{\mathrm{on}}}{d}; x_1\right),$<br>$A_2 = \frac{\mathrm{e}^{x_1\exp(-dt)}}{\sigma_{\mathrm{off}}+\sigma_{\mathrm{on}}} \sigma_{\mathrm{on}} M\left(1+\frac{\sigma_{\mathrm{on}}}{d}; 1+\frac{\sigma_{\mathrm{off}}+\sigma_{\mathrm{on}}}{d}; x_1\right),$<br>$A_3 = \frac{\mathrm{e}^{x_1\exp(-dt)}}{\sigma_{\mathrm{off}}+\sigma_{\mathrm{on}}} M\left(-\frac{\sigma_{\mathrm{off}}}{d}; 1-\frac{\sigma_{\mathrm{off}}+\sigma_{\mathrm{on}}}{d}; x_1\right),$<br>$A_4 = \frac{\mathrm{e}^{x_1\exp(-dt)}}{\sigma_{\mathrm{off}}+\sigma_{\mathrm{on}}} \sigma_{\mathrm{off}} M\left(\frac{\sigma_{\mathrm{on}}}{d}; 1+\frac{\sigma_{\mathrm{off}}+\sigma_{\mathrm{on}}}{d}; x_1\right),$<br>$B_1 = -\mathrm{e}^{-(\sigma_{\mathrm{off}}+\sigma_{\mathrm{on}})t} \sigma_{\mathrm{off}} M\left(\frac{\sigma_{\mathrm{on}}}{d}; 1+\frac{\sigma_{\mathrm{off}}+\sigma_{\mathrm{on}}}{d}; x_1\mathrm{e}^{-dt}\right),$<br>$B_2 = \mathrm{e}^{-(\sigma_{\mathrm{off}}+\sigma_{\mathrm{on}})t} \sigma_{\mathrm{on}} M\left(1+\frac{\sigma_{\mathrm{on}}}{d}; 1+\frac{\sigma_{\mathrm{off}}+\sigma_{\mathrm{on}}}{d}; x_1\mathrm{e}^{-dt}\right),$<br>$B_3 = M\left(-\frac{\sigma_{\mathrm{off}}}{d}; 1+\frac{\sigma_{\mathrm{off}}+\sigma_{\mathrm{on}}}{d}; x_1\mathrm{e}^{-dt}\right),$<br>$B_4 = M\left(1-\frac{\sigma_{\mathrm{off}}}{d}; 1-\frac{\sigma_{\mathrm{off}}+\sigma_{\mathrm{on}}}{d}; x_1\mathrm{e}^{-dt}\right)$	
Tele with feedback	$G + P \underset{\sigma_{\mathrm{on}}}{\overset{\sigma_{\mathrm{off}}}{\rightleftharpoons}} G^*,\ G \xrightarrow{\rho_u} G + P,$<br>$G^* \xrightarrow{\rho_b} G^* + P,\ P \xrightarrow{d} \varnothing$	SS	$\mathcal{G}(z_1) = A\mathrm{e}^{\frac{\rho_b u_1}{d}} \left(\frac{d^2\alpha + d\alpha\sigma_{\mathrm{off}}}{\rho_u\sigma_{\mathrm{off}}}\right) M(1+\alpha;\beta;\omega) + A\mathrm{e}^{\frac{\rho_b u_1}{d}} \left(1-\frac{d\alpha}{\rho_u-\rho_b}\right) M(\alpha;\beta;\omega),$<br>$A^{-1} = \left(\frac{d^2\alpha + d\alpha\sigma_{\mathrm{off}}}{\rho_u\sigma_{\mathrm{off}}}\right) M(1+\alpha;\beta;\omega_1) + \left(1-\frac{d\alpha}{\rho_u-\rho_b}\right) M(\alpha;\beta;\omega_1),$<br>$\alpha = (\sigma_{\mathrm{on}}\rho_u - \sigma_{\mathrm{on}}\rho_b)/(\rho_u d - \rho_b d - \rho_b\sigma_{\mathrm{off}})$<br>$\beta = 1 + (\sigma_{\mathrm{on}} d + \sigma_{\mathrm{off}}\sigma_{\mathrm{on}} + \sigma_{\mathrm{off}}\rho_u)/(d+\sigma_{\mathrm{off}})^2$<br>$\omega = (\rho_u d - \rho_b d - \rho_b\sigma_{\mathrm{off}})(du_1 + \sigma_{\mathrm{off}}u_1 + \sigma_{\mathrm{off}})/[d(d+\sigma_{\mathrm{off}})^2]$<br>$\omega_1 = (\rho_u d - \rho_b d - \rho_b\sigma_{\mathrm{off}})\sigma_{\mathrm{off}}/[d(d+\sigma_{\mathrm{off}})^2]$	[3]
Refractory	$G_1 \xrightarrow{\sigma_{\mathrm{b}}} G_2 \xrightarrow{\sigma_{\mathrm{u}}} G_3 \xrightarrow{\lambda} G_1,$<br>$G_1 \xrightarrow{\rho} G_1 + M,\ M \xrightarrow{d} \varnothing$	SS	$\mathcal{G}(z_1) = {}_2F_2\left(\frac{\lambda}{d}, \frac{\sigma_{\mathrm{u}}}{d}; v^-, v^+; x_1\right),$<br>$v^{\pm} = \frac{\lambda + \sigma_{\mathrm{b}} + \sigma_{\mathrm{u}}}{2d} \pm \frac{\sqrt{(\lambda-\sigma_{\mathrm{b}})^2 - 2(\lambda+\sigma_{\mathrm{b}})\sigma_{\mathrm{u}} + \sigma_{\mathrm{u}}^2}}{2d}$	[4]
Model with PRPR	$G_1 \xrightarrow{\sigma_{\mathrm{b}}} G_2 \underset{\sigma_{\mathrm{b}}}{\overset{\sigma_{\mathrm{u}}}{\rightleftharpoons}} G_3 \xrightarrow{\lambda} G_1,\ G_1 \xrightarrow{\rho} G_3 + M,\ M \xrightarrow{d} \varnothing$	SS	$\mathcal{G}(z_1) = {}_1F_2\left(\frac{\sigma_{\mathrm{u}}}{d}; \frac{\sigma_{\mathrm{b}}+\sigma_{\mathrm{u}}}{d}, \frac{\rho+\lambda+\sigma_{\mathrm{b}}}{d}; \frac{\lambda x_1}{d}\right)$	[4]

Model name	Reactions	TD/SS	PGF solution	Reference
Tele with feedback & bursty	$G \xrightarrow{\sigma_{\rm off}} G^*,\ G^* + P \xrightarrow{\sigma_{\rm on}} G + P,$ $G \xrightarrow{\rho_u f(b,i)} G + iP,\ G^* \xrightarrow{\rho_b f(b,i)} G^* + iP$ $P \xrightarrow{d} \varnothing.$	SS	$\mathcal{G}(z_1) = B(1-bu_1)^{-\frac{\rho_u}{d}}\, {}_2F_1\left(u, v; \mathbf{a}+1-w; 1-\phi(1-bu_1)\right),$ $B^{-1} = {}_2F_1\left(u, v; \mathbf{a}+1-w; 1-\phi\right),$ $u = (\mathbf{a} + \sqrt{\mathbf{a}^2 - 4\mathbf{b}})/2$ $v = (\mathbf{a} - \sqrt{\mathbf{a}^2 - 4\mathbf{b}})/2$ $\mathbf{a} = \frac{\rho_b - \rho_u + \sigma_{\rm off} - \sigma_{\rm on}\rho_u/d}{d + \sigma_{\rm on}}$ $\mathbf{b} = \frac{\sigma_{\rm off}(\rho_b - \rho_u)}{d(d+\sigma_{\rm on})}$ $w = \frac{\rho_b - \rho_u + d + \sigma_{\rm on}(1+b)(1-\rho_u/d)}{d + \sigma_{\rm on}(1+b)}$ $\phi = \frac{d + \sigma_{\rm on}}{d + \sigma_{\rm on} + b\sigma_{\rm on}}$	[5]
Three stage	$G_0 \underset{\sigma_{\rm on}}{\overset{\sigma_{\rm off}}{\rightleftharpoons}} G_1,\ G_0 \xrightarrow{\rho} G_0 + M,\ M \xrightarrow{\lambda} M + P,\ M \xrightarrow{d} \varnothing.$	TD	$\mathcal{G}(z_1, z_2, t) = \begin{bmatrix} 1 & 1 \end{bmatrix} \begin{bmatrix} k_1(u_1,u_2,t) & k_2(u_1,u_2,t) \\ k_3(u_1,u_2,t) & k_4(u_1,u_2,t) \end{bmatrix} \begin{bmatrix} g_0(\mathcal{T}_t u_1, u_2) \\ g_1(\mathcal{T}_t u_1, u_2) \end{bmatrix},$ $k_1(u_1,u_2,t) = \gamma_a \Big[-\epsilon_b\gamma_b M(\epsilon_a, r, x\mathrm{e}^{-st}) M(\epsilon_b+1, 2-r, x)$ $+ \epsilon_a M(\epsilon_b, 2-r, x\mathrm{e}^{-st}) M(\epsilon_a+1, r, x) \Big],$ $k_2(u_1,u_2,t) = \frac{\gamma_a\sigma_{\rm on}}{s} \Big[-\gamma_b M(\epsilon_a+1, r, x\mathrm{e}^{-st}) M(\epsilon_b+1, 2-r, x)$ $+ M(\epsilon_b+1, 2-r, x\mathrm{e}^{-st}) M(\epsilon_a+1, r, x) \Big],$ $k_3(u_1,u_2,t) = \frac{\gamma_a s\epsilon_a\epsilon_b}{\sigma_{\rm on}} \Big[\gamma_b M(\epsilon_a, r, x\mathrm{e}^{-st}) M(\epsilon_b, 2-r, x)$ $- M(\epsilon_b, 2-r, x\mathrm{e}^{-st}) M(\epsilon_a, r, x) \Big],$ $k_4(u_1,u_2,t) = \gamma_a \Big[\epsilon_a\gamma_b M(\epsilon_a+1, r, x\mathrm{e}^{-st}) M(\epsilon_b, 2-r, x)$ $- \epsilon_b M(\epsilon_b+1, 2-r, x\mathrm{e}^{-st}) M(\epsilon_a, r, x) \Big],$ $\gamma_a = \frac{\exp(kst - x\mathrm{e}^{-st})}{r-1},$ $\gamma_b = \exp[-st(r-1)],$ $v = du_1 - \lambda u_2 - \lambda u_1 u_2,$ $s = d - \lambda u_2,$ $x = \rho v/s^2,$ $\theta = \frac{d\rho}{s^2} - \frac{\rho + \sigma_{\rm off} - \sigma_{\rm on}}{s},$ $r = 1 + \sqrt{4\left(\frac{\rho d\sigma_{\rm on}}{s^3} - \frac{\rho\sigma_{\rm on}}{s^2}\right) + \left(\theta - \frac{2\sigma_{\rm on}}{s}\right)^2},$ $k = \left(\theta - 2\sigma_{\rm on}/s + r - 1\right)/2,$ $\mathcal{T}_t u_1 = \frac{(du_1 - \lambda u_2 - \lambda u_1 u_2)\mathrm{e}^{-st} + \lambda u_2}{d - \lambda u_2},$ $\epsilon_a = (r + \theta - 1)/2, \quad \epsilon_b = (-r + \theta + 1)/2$	[6]

Model name	Reactions	TD/SS	PGF solution	Reference
BD with DD	$G \xrightarrow{\rho} G+M,\ M \overset{\tau}{\Rightarrow} \varnothing$	SS	$\mathcal{G}(z_1) = \mathrm{e}^{\rho u_1 \tau}$	[7]
Bursty with DD	$G \xrightarrow{\rho f(b,i)} G+iM,\ M \overset{\tau}{\Rightarrow} \varnothing$	SS	$\mathcal{G}(z_1) = \exp\left(\frac{\rho b u_1 \tau}{1-bu_1}\right)$	[7]
Tele with DD	$G \underset{\sigma_{\mathrm{on}}}{\overset{\sigma_{\mathrm{off}}}{\rightleftharpoons}} G^*,\ G \xrightarrow{\rho} G+M,\ M \overset{\tau}{\Rightarrow} \varnothing$	SS	$\mathcal{G}(z_1) = \frac{\mu^+ \mathrm{e}^{-\mu^- \tau} - \mu^- \mathrm{e}^{-\mu^+ \tau}}{\theta} + \frac{\rho u_1 \sigma_{\mathrm{on}} (\mathrm{e}^{-\mu^- \tau} - \mathrm{e}^{-\mu^+ \tau})}{\theta(\sigma_{\mathrm{off}}+\sigma_{\mathrm{on}})},$ $\mu^\pm = (r \pm \theta - 1)/2,$ $\theta = \left[(\rho u_1 - \sigma_{\mathrm{off}} - \sigma_{\mathrm{on}})^2 + 4\rho\sigma_{\mathrm{on}} u_1\right]^{\frac{1}{2}},$ $r = 1 - \rho u_1 + \sigma_{\mathrm{off}} + \sigma_{\mathrm{on}}$	[8]
Two-compartment BD	$G \xrightarrow{\rho} G+N,\ N \overset{\tau}{\Rightarrow} M,\ M \xrightarrow{d} \varnothing$	SS	$\mathcal{G}(z_1, z_2) = \mathrm{e}^{\rho u_1 \tau + \rho u_2/d}$	[7]
Two-compartment bursty	$G \xrightarrow{\rho f(b,i)} G+iN,\ N \overset{\tau}{\Rightarrow} M,\ M \xrightarrow{d} \varnothing$	SS	$\mathcal{G}(z_1, z_2) = \exp\left(\frac{\rho b u_1 \tau}{1-bu_1}\right)(1-bu_2)^{-\frac{\rho}{d}}$	[7]
Two-compartment Tele	$G \underset{\sigma_{\mathrm{on}}}{\overset{\sigma_{\mathrm{off}}}{\rightleftharpoons}} G^*,\ G \xrightarrow{\rho} G+N,$ $N \overset{\tau}{\Rightarrow} M,\ M \xrightarrow{d} \varnothing$	SS	$\mathcal{G}(z_1, z_2) = \frac{\mu_+ e^{-\mu_- \tau} - \mu_- e^{-\mu_+ \tau}}{d\theta} M\left(\frac{\sigma_{\mathrm{on}}}{d}, \frac{\sigma_{\mathrm{off}}+\sigma_{\mathrm{on}}}{d}, x_2\right) + \frac{\rho u_1 \sigma_{\mathrm{on}} (e^{-\mu_- \tau} - e^{-\mu_+ \tau})}{d\theta(\sigma_{\mathrm{off}}+\sigma_{\mathrm{on}})} M\left(1+\frac{\sigma_{\mathrm{on}}}{d}, 1+\frac{\sigma_{\mathrm{off}}+\sigma_{\mathrm{on}}}{d}, x_2\right)$ $\mu^\pm = d(r \pm \theta - 1)/2,$ $\theta = \left[(x_1 - \sigma_{\mathrm{off}}/d - \sigma_{\mathrm{on}}/d)^2 + 4\sigma_{\mathrm{on}} x_1/d\right]^{\frac{1}{2}},$ $r = 1 - x_1 + (\sigma_{\mathrm{off}}+\sigma_{\mathrm{on}})/d$	[8]
Two-compartment refractory	$G_1 \xrightarrow{\sigma_{\mathrm{b}}} G_2 \xrightarrow{\sigma_{\mathrm{u}}} G_3 \xrightarrow{\lambda} G_1,$ $G_1 \xrightarrow{\rho} G_1+N,\ N \overset{\tau}{\Rightarrow} M,\ M \xrightarrow{d} \varnothing$	SS	$\mathcal{G}(z_1, z_2) = {}_2F_2\left(\frac{\lambda}{d}, \frac{\sigma_{\mathrm{u}}}{d}; v^-, v^+; x_2\right) + j_{12} F_2\left(\frac{\lambda}{d}, \frac{\sigma_{\mathrm{u}}}{d}; v^-+1, v^++1; x_2\right) + j_{22} F_2\left(\frac{\lambda}{d}+1, \frac{\sigma_{\mathrm{u}}}{d}; v^-+1, v^++1; x_2\right) + j_{32} F_2\left(\frac{\lambda}{d}+1, \frac{\sigma_{\mathrm{u}}}{d}+1; v^-+1, v^++1; x_2\right),$ $j_1 = -\frac{\sigma_{\mathrm{b}}\sigma_{\mathrm{u}}}{\mu_0} + \frac{\mathrm{e}^{r_1\tau} x_1 \lambda \sigma_{\mathrm{b}} \sigma_{\mathrm{u}} (r_1+\sigma_{\mathrm{u}})}{r_1 \mathcal{B} \mu_0} - \frac{\mathrm{e}^{a\tau} x_1 \lambda \sigma_{\mathrm{b}} \sigma_{\mathrm{u}} \left[\mathcal{J} + (2a - r_1)\sigma_{\mathrm{u}}\right] \cos(b\tau)}{\mathcal{B}\mathcal{J}\mu_0} + \frac{\mathrm{e}^{a\tau} x_1 \lambda \sigma_{\mathrm{b}} \sigma_{\mathrm{u}} \left[\mathcal{J}(a-r_1) + (a^2 - b^2 - ar_1)\sigma_{\mathrm{u}}\right] \sin(b\tau)}{b\mathcal{B}\mathcal{J}\mu_0},$ $j_2 = -\frac{\lambda\sigma_{\mathrm{b}}}{\mu_0} + \frac{\mathrm{e}^{r_1\tau} x_1 \lambda^2 \sigma_{\mathrm{b}} \sigma_{\mathrm{u}}}{r_1 \mathcal{B} \mu_0} - \frac{\mathrm{e}^{a\tau} x_1 \lambda^2 \sigma_{\mathrm{b}} \sigma_{\mathrm{u}} (2a - r_1) \cos(b\tau)}{\mathcal{B}\mathcal{J}\mu_0} + \frac{\mathrm{e}^{a\tau} x_1 \lambda^2 \sigma_{\mathrm{b}} \sigma_{\mathrm{u}} (a^2 - ar_1 - b^2) \sin(b\tau)}{b\mathcal{B}\mathcal{J}\mu_0},$ $j_3 = -\frac{\lambda\sigma_{\mathrm{u}}}{\mu_0} + \frac{\mathrm{e}^{r_1\tau} x_1 \lambda \sigma_{\mathrm{u}} (r_1+\lambda)(r_1+\sigma_{\mathrm{u}})}{r_1 \mathcal{B} \mu_0} - \frac{\mathrm{e}^{a\tau} x_1 \lambda \sigma_{\mathrm{u}} \left[\mathcal{J}(r_1+\lambda+\sigma_{\mathrm{u}}) + (2a - r_1)\lambda\sigma_{\mathrm{u}}\right] \cos(b\tau)}{\mathcal{B}\mathcal{J}\mu_0} + \frac{\mathrm{e}^{a\tau} x_1 \lambda \sigma_{\mathrm{u}} \left[\mathcal{J}(b^2 + (a-r_1)(a+\lambda)) + \mathcal{J}(a-r_1)\sigma_{\mathrm{u}} + (a^2 - b^2 - ar_1)\lambda\sigma_{\mathrm{u}}\right] \sin(b\tau)}{b\mathcal{B}\mathcal{J}\mu_0},$ $\mathcal{J} = a^2 + b^2, \quad \mathcal{B} = (a-r_1)^2 + b^2,$ $v^\pm = \frac{\lambda + \sigma_{\mathrm{b}} + \sigma_{\mathrm{u}}}{2d} \pm \frac{\sqrt{(\lambda-\sigma_{\mathrm{b}})^2 - 2(\lambda+\sigma_{\mathrm{b}})\sigma_{\mathrm{u}} + \sigma_{\mathrm{u}}^2}}{2d}$	[8]

A. Properties of probability generating functions

(P1) *Binomial partitioning:* Given that each random variable m_i in the vector $\mathbf{m}$ is independently drawn from the corresponding random variable n_i in the vector $\mathbf{n}$ with a binomial distribution, the probability mass function for m_i given n_i is:

$$P(m_i|n_i) = \binom{n_i}{m_i} p^{m_i}(1-p)^{n_i-m_i},$$

where p is the success probability in the binomial distribution. The PGF $\bar{G}(\mathbf{z})$ for the random vector $\mathbf{m}$ can be derived using the relationship between the PGFs of $\mathbf{n}$ and $\mathbf{m}$. The PGF of $\mathbf{m}$ is given by:

$$\bar{G}(\mathbf{z}) = G(p\mathbf{z} + (1-p)\mathbf{1}),$$

where $\mathbf{1} = [1, \cdots, 1]^\top$. This transformation reflects the fact that for a binomial distribution, the generating function of m_i given n_i is $(pz_i + 1 - p)^{n_i}$. Applying this transformation to each component of $\mathbf{z}$ in the original PGF yields the PGF for the vector $\mathbf{m}$. See the supplementary information of Ref. [9] for a detailed proof. This process closely resembles the partitioning of molecules between daughter cells during cell division, where each molecule in the mother cell has a certain probability of being allocated to one daughter cell.

(P2) *Marginalization:* The PGF marginalized with respect to the random variable n_i is obtained by setting $z_i = 1$ in the PGF. Thus, the marginalized PGF is expressed as $G(\mathbf{z})|_{z_i=1}$. This operation effectively removes the dependence on the random variable n_i, resulting in the PGF for the distribution of the remaining variables.

(P3) *Summation of all variables:* Given that $g(z)$ is the PGF for the random variable $n = \sum_{i=1}^{N} n_i$, the PGF $g(z)$ is given by

$$g(z) = G(z\mathbf{1}).$$

This expression indicates that the PGF of the sum of the random variables n_i is obtained by evaluating the original PGF $G(\mathbf{z})$ at the point where each component z_i of the vector $\mathbf{z}$ is replaced by the same value z. This can be seen from

$$g(z) = \langle z^n \rangle = \left\langle z^{\sum_{i=1}^{N} n_i} \right\rangle = \left\langle \prod_{i=1}^{N} z^{n_i} \right\rangle = G(z\mathbf{1}).$$

(P4) *Independence:* Given two random vectors $\mathbf{n}, \mathbf{m} \in \mathbb{N}^N$, with their respective PGFs $G_1(\mathbf{z}_1)$ and $G_2(\mathbf{z}_2)$, if each random variable n_i in $\mathbf{n}$ is independent of m_j in $\mathbf{m}$ for any i and j, the joint PGF of $\mathbf{n}$ and $\mathbf{m}$ is given by

$$G(\mathbf{z}_1, \mathbf{z}_2) = G_1(\mathbf{z}_1)G_2(\mathbf{z}_2).$$

This means that the joint PGF of two independent random vectors is simply the product of their individual PGFs. Consider a new random vector $\mathbf{q} = \mathbf{n} + \mathbf{m}$. Its corresponding PGF, $\bar{G}(\mathbf{z})$, can be directly obtained by applying properties P1 and P4, resulting in the following expression:

$$\bar{G}(\mathbf{z}) = G_1(\mathbf{z})G_2(\mathbf{z}).$$

(P5) *Zero inflation:* Given a random vector $\mathbf{n} \in \mathbb{N}^N$ with probability distribution $P(\mathbf{n})$ and PGF $G(\mathbf{z})$, consider a new random vector $\mathbf{m} \in \mathbb{N}^N$ with the probability distribution defined as

$$P(\mathbf{m}) = \lambda P(\mathbf{n}) + (1-\lambda)\delta_{\mathbf{m}=\mathbf{0}}.$$

Here $\delta_{\mathbf{m}=\mathbf{0}}$ is the Dirac delta function, which equals 1 if $\mathbf{m}$ is the all-zero vector $\mathbf{0} = [0, \cdots, 0]^\top$, and 0 otherwise. The PGF of $\mathbf{m}$ is then given by

$$\bar{G}(\mathbf{z}) = \lambda G(\mathbf{z}) + 1 - \lambda.$$

This model is commonly used to describe technical errors encountered during the analysis of single-cell RNA sequencing datasets.

Additionally, the linear mapping approximation (LMA) [10] and its variant [11] allow us to derive PGF solutions for more complex reaction systems using the PGF solutions listed in Table A.

B. Exact time-dependent solution of the refractory model

For the three-state refractory model illustrated in the inset of Fig 4A, the corresponding CMEs are given by

$$\begin{cases} \dfrac{\mathrm{d}P_1(n,t)}{\mathrm{d}t} = d(\mathbb{E}-1)nP_1(n,t) + \rho(\mathbb{E}^{-1}-1)P_1(n,t) - \sigma_{\mathrm{b}}P_1(n,t) \\ \qquad\qquad + \lambda P_3(n,t), \\ \dfrac{\mathrm{d}P_2(n,t)}{\mathrm{d}t} = \sigma_{\mathrm{b}}P_1(n,t) - \sigma_{\mathrm{u}}P_2(n,t) + d(\mathbb{E}-1)nP_2(n,t), \\ \dfrac{\mathrm{d}P_3(n,t)}{\mathrm{d}t} = \sigma_{\mathrm{u}}P_2(n,t) - \lambda P_3(n,t) + d(\mathbb{E}-1)nP_3(n,t), \end{cases} \tag{S1}$$

where $P_\phi(n,t)$ denotes the probability of observing n mRNA molecules when the gene is in state $\phi \in \{1,2,3\}$.

Defining the generating function $G_\phi \sum_n (u+1)^n P_\phi(n,t)$, Eq. (S1) can be recast as the following system of PDEs

$$\begin{cases} \partial_t G_1 = -\sigma_{\mathrm{b}}G_1 + \lambda G_3 + \rho u G_1 - du\partial_u G_1, & \text{(S2a)} \\ \partial_t G_2 = \sigma_{\mathrm{b}}G_1 - \sigma_{\mathrm{u}}G_2 - du\partial_u G_2, & \text{(S2b)} \\ \partial_t G_3 = \sigma_{\mathrm{u}}G_2 - \lambda G_3 - du\partial_u G_3. & \text{(S2c)} \end{cases}$$

We first use Eq. (S2b) to express G_2 in terms of G_1, and subsequently apply Eq. (S2c) to represent G_3 in terms of G_2,

$$\begin{cases} G_1 = \dfrac{\sigma_{\mathrm{u}}G_2 + du\partial_u G_2 + \partial_t G_2}{\sigma_{\mathrm{b}}}, & \text{(S3a)} \\ G_2 = \dfrac{\lambda G_3 + du\partial_u G_3 + \partial_t G_3}{\sigma_{\mathrm{u}}}. & \text{(S3b)} \end{cases}$$

Substituting Eq. (S3b) into Eq. (S3a) and simplifying yields an explicit expression for G_1 in terms of G_3,

$$G_1 = \frac{1}{\sigma_{\mathrm{b}}\sigma_{\mathrm{u}}}[\partial^2_{tt}G_3 + 2du\partial^2_{tu}G_3 + d^2u^2\partial^2_{uu}G_3 + (\lambda+\sigma_{\mathrm{u}})\partial_t G_3 \\ + (d+\lambda+\sigma_{\mathrm{u}})du\partial_u G_3 + \lambda\sigma_{\mathrm{u}}G_3]. \tag{S4}$$

Summing Eqs. (S2a)–(S2c) and substituting Eq. (S4) into the result produces a single parabolic PDE for G_3,

$$\begin{aligned} & d^3u^3\partial^3_{uuu}G_3 + 3d^2u^2\partial^3_{uut}G_3 + 3du\partial^3_{utt}G_3 + \partial^3_{ttt}G_3 - \rho u\lambda\sigma_{\mathrm{u}}G_3 \\ & + d^2u^2(3d+\lambda+\sigma_{\mathrm{u}}+\sigma_{\mathrm{b}}-\rho u)\partial^2_{uu}G_3 + (\lambda+\sigma_{\mathrm{u}}+\sigma_{\mathrm{b}}-\rho u)\partial^2_{tt}G_3 \\ & + du(3d+2\lambda+2\sigma_{\mathrm{u}}+2\sigma_{\mathrm{b}}-2\rho u)\partial^2_{ut}G_3 \\ & + du[(d+\lambda)(d+\sigma_{\mathrm{u}}+\sigma_{\mathrm{b}}) + \sigma_{\mathrm{u}}\sigma_{\mathrm{b}} - \rho u(d+\lambda+\sigma_{\mathrm{u}})]\partial_u G_3 \\ & + [(\lambda\sigma_{\mathrm{b}}+\lambda\sigma_{\mathrm{u}}+\sigma_{\mathrm{b}}\sigma_{\mathrm{u}}) - \rho u(\lambda+\sigma_{\mathrm{u}})]\partial_t G_3 = 0. \end{aligned} \tag{S5}$$

To solve Eq. (S5), we introduce the change of variables $(t,u) \mapsto (w,v)$ with $v = du$ and $w = \ln du - dt$. This transformation can be implemented in `Mathematica` using the `DSolvechangeVariables` option. After simplification, the PDE reduces to a third-order ODE,

$$\begin{aligned} & v^2\partial^3_{vvv}G_3 + \left(3 + \frac{\lambda+\sigma_{\mathrm{b}}+\sigma_{\mathrm{u}}}{d} - \frac{\rho v}{d^2}\right)v\partial^2_{vv}G_3 - \frac{\rho\lambda\sigma_{\mathrm{u}}}{d^4}G_3 \\ & + \left[1 + \frac{\lambda+\sigma_{\mathrm{b}}+\sigma_{\mathrm{u}}}{d} + \frac{\sigma_{\mathrm{b}}\sigma_{\mathrm{u}}+\lambda\sigma_{\mathrm{b}}+\lambda\sigma_{\mathrm{u}}}{d^2} - \frac{\rho v(d+\lambda+\sigma_{\mathrm{u}})}{d^3}\right]\partial_v G_3 = 0. \end{aligned} \tag{S6}$$

Changing to a description in terms of the variable $x = \rho v/d^2$, Eq. (S6) further simplifies to

$$\begin{aligned} & x^2\partial^3_{xxx}G_3 + \left(3 + \frac{\lambda+\sigma_{\mathrm{b}}+\sigma_{\mathrm{u}}}{d} - x\right)x\partial^2_{xx}G_3 - \frac{\lambda\sigma_{\mathrm{u}}}{d^2}G_3 \\ & + \left[1 + \frac{\lambda+\sigma_{\mathrm{b}}+\sigma_{\mathrm{u}}}{d} + \frac{\sigma_{\mathrm{b}}\sigma_{\mathrm{u}}+\lambda\sigma_{\mathrm{b}}+\lambda\sigma_{\mathrm{u}}}{d^2} - \left(1 + \frac{\lambda}{d} + \frac{\sigma_{\mathrm{u}}}{d}\right)x\right]\partial_x G_3 = 0. \end{aligned} \tag{S7}$$

This corresponds to the canonical form of the generalized hypergeometric differential equation (see Eq. (16.8.3) in [12]), from which it follows that the general solution of Eq. (S7) is

$$G_3 = C_0(J)W_0 + C_1(J)W_1 + C_2(J)W_2. \tag{S8}$$

where $J = \exp(w)$. The functions W_0, W_1, and W_2 are defined as follows

$$\begin{cases} W_0 = \mathbf{F}^0_{x1}, \\ W_1 = x^{-(k-\Delta)/2d}\mathbf{F}^0_{x2}, \\ W_2 = x^{-(k+\Delta)/2d}\mathbf{F}^0_{x3}, \end{cases} \tag{S9}$$

Here $k = \lambda + \sigma_\mathrm{b} + \sigma_\mathrm{u}$ and $\Delta = \sqrt{(\lambda - \sigma_\mathrm{b})^2 - 2(\lambda + \sigma_\mathrm{b})\sigma_\mathrm{u} + \sigma_\mathrm{u}^2}$. The functions $\mathbf{F}^i_{x1}$, $\mathbf{F}^i_{x2}$, and $\mathbf{F}^i_{x3}$ are expressed in terms of generalized hypergeometric functions as

$$\begin{aligned} \mathbf{F}^i_{x1} &= {}_2F_2\left(i + \frac{\lambda}{d}, i + \frac{\sigma_\mathrm{u}}{d}; i + 1 + \frac{k}{2d} - \frac{\Delta}{2d}, i + 1 + \frac{k}{2d} + \frac{\Delta}{2d}; x\right), \\ \mathbf{F}^i_{x2} &= {}_2F_2\left(i + \frac{\lambda}{d} - \frac{k}{2d} + \frac{\Delta}{2d}, i + \frac{\sigma_\mathrm{u}}{d} - \frac{k}{2d} + \frac{\Delta}{2d}; i + 1 - \frac{k}{2d} + \frac{\Delta}{2d}, i + 1 + \frac{\Delta}{d}; x\right), \\ \mathbf{F}^i_{x3} &= {}_2F_2\left(i + \frac{\lambda}{d} - \frac{k}{2d} - \frac{\Delta}{2d}, i + \frac{\sigma_\mathrm{u}}{d} - \frac{k}{2d} - \frac{\Delta}{2d}; i + 1 - \frac{k}{2d} - \frac{\Delta}{2d}, i + 1 - \frac{\Delta}{d}; x\right). \end{aligned} \tag{S10}$$

Note that the coefficients C_0, C_1, and C_2 in Eq. (S8) are functions of $J = \exp(w)$, and thus depend only on w. Their specific forms are determined by the initial conditions.

To this end, we first express G_1 and G_2 in terms of C_0, C_1, C_2, and the basis functions W_0, W_1, and W_2 using Eqs. (S4) and (S3b). With the substitutions $v = du$ and $J = du\exp(-dt)$, the resulting forms of G_1 and G_2 are

$$\begin{cases} G_2 = \dfrac{\lambda}{\sigma_\mathrm{u}}(C_0W_0 + C_1W_1 + C_2W_2) + \dfrac{dx}{\sigma_\mathrm{u}}(C_0W_0' + C_1W_1' + C_2W_2'), \\ G_1 = \dfrac{\lambda}{\sigma_\mathrm{b}}(C_0W_0 + C_1W_1 + C_2W_2) + \dfrac{(d + \lambda + \sigma_\mathrm{u})dx}{\sigma_\mathrm{b}\sigma_\mathrm{u}}(C_0W_0' \\ \qquad + C_1W_1' + C_2W_2') + \dfrac{d^2x^2}{\sigma_\mathrm{b}\sigma_\mathrm{u}}(C_0W_0'' + C_1W_1'' + C_2W_2''), \end{cases} \tag{S11}$$

where W_0', W_1', and W_2' denote the derivatives of W_0, W_1, and W_2 with respect to x, given explicitly by

$$\begin{aligned} W_0' &= \frac{4\lambda\sigma_\mathrm{u}}{(2d + k - \Delta)(2d + k + \Delta)}\mathbf{F}^1_{x1}, \\ W_1' &= \left(-\frac{k}{2d} + \frac{\Delta}{2d}\right)x^{(-k+\Delta)/2d-1}\mathbf{F}^0_{x2} + \frac{(2\lambda - k + \Delta)(2\sigma_\mathrm{u} - k + \Delta)}{(2d - k + \Delta)(2d + 2\Delta)}x^{(-k+\Delta)/2d}\mathbf{F}^1_{x2}, \\ W_2' &= \left(-\frac{k}{2d} - \frac{\Delta}{2d}\right)x^{(-k-\Delta)/2d-1}\mathbf{F}^0_{x3} + \frac{(2\lambda - k - \Delta)(2\sigma_\mathrm{u} - k - \Delta)}{(2d - k - \Delta)(2d - 2\Delta)}x^{(-k-\Delta)/2d}\mathbf{F}^1_{x3}. \end{aligned} \tag{S12}$$

Similarly, W_0'', W_1'', and W_2'' denote the second derivatives of W_0, W_1, and W_2 with respect to x, and are given by

$$\begin{aligned}
W_0'' =& \frac{16\lambda\sigma_{\mathrm{u}}(d+\lambda)(d+\sigma_{\mathrm{u}})}{(2d+k-\Delta)(2d+k+\Delta)(4d+k-\Delta)(4d+k+\Delta)}\mathbf{F}_{x1}^2, \\
W_1'' =& \frac{(2\lambda-k+\Delta)(2\sigma_{\mathrm{u}}-k+\Delta)}{(2d-k+\Delta)(d+\Delta)}\left(-\frac{k}{2d}+\frac{\Delta}{2d}\right)x^{(-k+\Delta)/2d-1}\mathbf{F}_{x2}^1 \\
&+\left(-\frac{k}{2d}+\frac{\Delta}{2d}\right)\left(-\frac{k}{2d}+\frac{\Delta}{2d}-1\right)x^{(-k+\Delta)/2d-2}\mathbf{F}_{x2}^0 \\
&+\frac{(2\lambda-k+\Delta)(2\sigma_{\mathrm{u}}-k+\Delta)}{(2d-k+\Delta)(2d+2\Delta)}\frac{(2d+2\lambda-k+\Delta)(2d+2\sigma_{\mathrm{u}}-k+\Delta)}{(4d-k+\Delta)(4d+2\Delta)}, \\
&\times x^{(-k+\Delta)/2d}\mathbf{F}_{x2}^2 \\
W_2'' =& \frac{(2\lambda-k-\Delta)(2\sigma_{\mathrm{u}}-k-\Delta)}{(2d-k-\Delta)(d-\Delta)}\left(-\frac{k}{2d}-\frac{\Delta}{2d}\right)x^{(-k-\Delta)/2d-1}\mathbf{F}_{x3}^1 \\
&+\left(-\frac{k}{2d}-\frac{\Delta}{2d}\right)\left(-\frac{k}{2d}-\frac{\Delta}{2d}-1\right)x^{(-k-\Delta)/2d-2}\mathbf{F}_{x3}^0 \\
&+\frac{(2\lambda-k-\Delta)(2\sigma_{\mathrm{u}}-k-\Delta)}{(2d-k-\Delta)(2d-2\Delta)}\frac{(2d+2\lambda-k-\Delta)(2d+2\sigma_{\mathrm{u}}-k-\Delta)}{(4d-k-\Delta)(4d-2\Delta)} \\
&\times x^{(-k-\Delta)/2d}\mathbf{F}_{x3}^2.
\end{aligned} \tag{S13}$$

Without loss of generality, we assume that the gene starts in state G_1 with no mRNA transcripts. Equivalently, the initial conditions are $P_\phi(n,0)=0$ for all $\phi\in\{1,2,3\}$ and $n\geq 1$ together with $P_2(0,0)=P_3(0,0)=0$ and $P_1(0,0)=1$. At $t=0$, we have $J=v$. Using the initial conditions together with Eqs. (S8) and (S11), the coefficients C_0, C_1, and C_2 can be determined from

$$\begin{cases}
C_0W_0+C_1W_1+C_2W_2=0, \\
\dfrac{\lambda}{\sigma_{\mathrm{u}}}(C_0W_0+C_1W_1+C_2W_2)+\dfrac{dx}{\sigma_{\mathrm{u}}}(C_0W_0'+C_1W_1'+C_2W_2')=0, \\
\dfrac{\lambda}{\sigma_{\mathrm{b}}}(C_0W_0+C_1W_1+C_2W_2)+\dfrac{(d+\lambda+\sigma_{\mathrm{u}})dx}{\sigma_{\mathrm{b}}\sigma_{\mathrm{u}}}(C_0W_0'+C_1W_1' \\
+C_2W_2')+\dfrac{d^2x^2}{\sigma_{\mathrm{b}}\sigma_{\mathrm{u}}}(C_0W_0''+C_1W_1''+C_2W_2'')=1,
\end{cases} \tag{S14}$$

which leads to

$$\begin{cases}
C_0=\dfrac{(W_1'W_2-W_1W_2')\sigma_{\mathrm{b}}\sigma_{\mathrm{u}}}{d^2x^2\gamma}, \\
C_1=\dfrac{(W_2'W_0-W_2W_0')\sigma_{\mathrm{b}}\sigma_{\mathrm{u}}}{d^2x^2\gamma}, \\
C_2=\dfrac{(W_0'W_1-W_0W_1')\sigma_{\mathrm{b}}\sigma_{\mathrm{u}}}{d^2x^2\gamma}.
\end{cases} \tag{S15}$$

Here

$$\gamma=-\begin{vmatrix} W_0 & W_1 & W_2 \\ W_0' & W_1' & W_2' \\ W_0'' & W_1'' & W_2'' \end{vmatrix}=-\mathfrak{W}(x), \tag{S16}$$

where $\mathfrak{W}$ denotes the Wronskian identity, depending on W_0, W_1, and W_2, and expressed as a function of x. According to Abel's identity [13] and Eq. (S7), the following relation can be established for the Wronskian identity,

$$\begin{aligned}
\mathfrak{W}(x)&=\mathfrak{W}(0)\mathrm{exp}\left(-\int_0^x\frac{3d+\lambda+\sigma_b+\sigma_u}{dx}-1\mathrm{d}x\right) \\
&=\mathfrak{W}(0)\mathrm{e}^x x^{-3-k/d}.
\end{aligned} \tag{S17}$$

As per the definition of hypergeometric function (see Eq. (16.2.1) in Ref. [12])

$$ {}_2F_2(a_1,a_2;b_1,b_2;x)=\sum_{k=0}^{\infty}\frac{(a_1)_k(a_2)_k}{(b_1)_k(b_2)_k}\frac{x^k}{k!}, $$

the following limits can be derived

$$ \begin{aligned} &\lim_{x\to 0}W_0\sim 1+O(x),\\ &\lim_{x\to 0}W_1\sim x^{(-k+\Delta)/2d}[1+O(x)],\\ &\lim_{x\to 0}W_2\sim x^{(-k-\Delta)/2d}[1+O(x)]. \end{aligned} \tag{S18} $$

This further leads to

$$ \begin{aligned} \mathfrak{W}(0)&=\lim_{x\to 0}\mathfrak{W}(x)\mathrm{e}^{-x}x^{3+k/d}\\ &=\lim_{x\to 0}-\mathrm{e}^{-x}x^{3+k/d}\begin{vmatrix}1 & D_1 & D_2\\ 0 & D_3 & D_3\\ 0 & D_5 & D_6\end{vmatrix}\\ &=\lim_{x\to 0}-\mathrm{e}^{-x}\frac{(\sigma_b\sigma_u+\lambda\sigma_b+\lambda\sigma_u)\Delta}{d^3}\\ &=-\frac{(\sigma_b\sigma_u+\lambda\sigma_b+\lambda\sigma_u)\Delta}{d^3}, \end{aligned} \tag{S19} $$

where

$$ \begin{aligned} D_1&=x^{(-k+\Delta)/2d},\\ D_2&=x^{(-k-\Delta)/2d},\\ D_3&=\frac{-k+\Delta}{2d}x^{(-k+\Delta)/2d-1},\\ D_4&=\frac{-k-\Delta}{2d}x^{(-k-\Delta)/2d-1},\\ D_5&=\frac{(-k+\Delta)(-k+\Delta-2d)}{4d^2}x^{(-k+\Delta)/2d-2},\\ D_6&=\frac{(-k-\Delta)(-k-\Delta-2d)}{4d^2}x^{(-k-\Delta)/2d-2}. \end{aligned} $$

Therefore, it concludes from Eqs. (S16), (S19) and (S17) that

$$ \gamma=\frac{(\sigma_{\mathrm{b}}\sigma_{\mathrm{u}}+\lambda\sigma_{\mathrm{b}}+\lambda\sigma_{\mathrm{u}})\Delta}{d^3}x^{-3-k/d}\exp(x). \tag{S20} $$

From Eq. (S8), the coefficients C_0, C_1, and C_2 in Eq. (S15) are functions of the variable J, where $J=ve^{-dt}$ reduces to v at $t=0$. Thus, to fully characterize C_0, C_1, and C_2 for arbitrary t, we replace v with J, i.e., $v\mapsto ve^{-dt}$ and $x\mapsto xe^{-dt}$, obtaining

$$ \begin{aligned} C_0=&\frac{\sigma_{\mathrm{b}}\sigma_{\mathrm{u}}\mathrm{e}^{-h}}{\theta}\mathbf{F}^0_{h2}\mathbf{F}^0_{h3}+\frac{(2\lambda-k+\Delta)(2\sigma_{\mathrm{u}}-k+\Delta)\sigma_{\mathrm{b}}\sigma_{\mathrm{u}}dh\mathrm{e}^{-h}}{(2d-k+\Delta)(2d+2\Delta)\theta\Delta}\mathbf{F}^1_{h2}\mathbf{F}^0_{h3}\\ &-\frac{(2\lambda-k-\Delta)(2\sigma_{\mathrm{u}}-k-\Delta)\sigma_{\mathrm{b}}\sigma_{\mathrm{u}}dh\mathrm{e}^{-h}}{(2d-k-\Delta)(2d-2\Delta)\theta\Delta}\mathbf{F}^0_{h2}\mathbf{F}^1_{h3},\\ C_1=&-\frac{(k+\Delta)\sigma_{\mathrm{b}}\sigma_{\mathrm{u}}h^{(-\Delta+k)/2d}\mathrm{e}^{-h}}{2\theta\Delta}\mathbf{F}^0_{h1}\mathbf{F}^0_{h3}-\frac{4\lambda\sigma_{\mathrm{b}}\sigma_{\mathrm{u}}^2dh^{(-\Delta+k)/2d+1}\mathrm{e}^{-h}}{(2d+k-\Delta)(2d+k+\Delta)\theta\Delta}\\ &\times\mathbf{F}^1_{h1}\mathbf{F}^0_{h3}+\frac{(2\lambda-k-\Delta)(2\sigma_{\mathrm{u}}-k-\Delta)\sigma_{\mathrm{b}}\sigma_{\mathrm{u}}dh^{(-\Delta+k)/2d+1}\mathrm{e}^{-h}}{(2d-k-\Delta)(2d-2\Delta)\theta\Delta}\mathbf{F}^0_{h1}\mathbf{F}^1_{h3},\\ C_2=&\frac{(k-\Delta)\sigma_{\mathrm{b}}\sigma_{\mathrm{u}}h^{(\Delta+k)/2d}\mathrm{e}^{-h}}{2\theta\Delta}\mathbf{F}^0_{h1}\mathbf{F}^0_{h2}-\frac{4\lambda\sigma_{\mathrm{b}}\sigma_{\mathrm{u}}^2dh^{(\Delta+k)/2d+1}\mathrm{e}^{-h}}{(2d+k-\Delta)(2d+k+\Delta)\theta\Delta}\\ &\times\mathbf{F}^1_{h1}\mathbf{F}^0_{h2}-\frac{(2\lambda-k+\Delta)(2\sigma_{\mathrm{u}}-k+\Delta)\sigma_{\mathrm{b}}\sigma_{\mathrm{u}}dh^{(\Delta+k)/2d+1}\mathrm{e}^{-h}}{(2d-k+\Delta)(2d+2\Delta)\theta\Delta}\mathbf{F}^0_{h1}\mathbf{F}^1_{h2}, \end{aligned} \tag{S21} $$

where $h = xe^{-dt}$ and $\theta = \sigma_{\rm b}\sigma_{\rm u} + \lambda\sigma_{\rm b} + \lambda\sigma_{\rm u}$. The functions $\mathbf{F}^i_{h1}$, $\mathbf{F}^i_{h2}$, and $\mathbf{F}^i_{h3}$ are expressed in terms of generalized hypergeometric functions as

$$\begin{aligned}
\mathbf{F}^i_{h1} &= {}_2F_2\left(i+\frac{\lambda}{d}, i+\frac{\sigma_{\rm u}}{d}; i+1+\frac{k}{2d}-\frac{\Delta}{2d}, i+1+\frac{k}{2d}+\frac{\Delta}{2d}; h\right),\\
\mathbf{F}^i_{h2} &= {}_2F_2\left(i+\frac{\lambda}{d}-\frac{k}{2d}+\frac{\Delta}{2d}, i+\frac{\sigma_{\rm u}}{d}-\frac{k}{2d}+\frac{\Delta}{2d}; i+1-\frac{k}{2d}+\frac{\Delta}{2d}, i+1+\frac{\Delta}{d}; h\right),\\
\mathbf{F}^i_{h3} &= {}_2F_2\left(i+\frac{\lambda}{d}-\frac{k}{2d}-\frac{\Delta}{2d}, i+\frac{\sigma_{\rm u}}{d}-\frac{k}{2d}-\frac{\Delta}{2d}; i+1-\frac{k}{2d}-\frac{\Delta}{2d}, i+1-\frac{\Delta}{d}; h\right).
\end{aligned} \tag{S22}$$

Finally, combining Eqs. (S8) and (S11), the time-dependent solution of the refractory model, initialized in state G_1 with zero mRNA transcripts, is given by

$$\begin{aligned}
G =& \frac{\sigma_{\rm b}\sigma_{\rm u} + \lambda\sigma_{\rm b} + \lambda\sigma_{\rm u}}{\sigma_{\rm b}\sigma_{\rm u}}(C_0W_0 + C_1W_1 + C_2W_2)\\
&+ \frac{(d+\lambda+\sigma_{\rm b}+\sigma_{\rm u})dx}{\sigma_{\rm b}\sigma_{\rm u}}(C_0W_0' + C_1W_1' + C_2W_2')\\
&+ \frac{d^2x^2}{\sigma_{\rm b}\sigma_{\rm u}}(C_0W_0'' + C_1W_1'' + C_2W_2''),
\end{aligned} \tag{S23}$$

together with Eqs. (S9), (S12), (S13), (S21) and (S22).

TABLE B. Parameter values used in Fig 2.

Set	ρ	σ_{on}	σ_{off}
1	9.64	2.76	1.66
2	8.76	0.97	0.19
3	6.26	2.86	1.00
4	7.24	2.32	0.80
5	8.54	2.60	2.69

TABLE C. Ground-truth kinetic parameters and parameter estimates for Fig 4B and 4C.

Models	ρ	σ_b	σ_u	λ	d
Refractory (ground-truth)	9	1	0.5	0.6	1
Refractory (Fig 4B)	8.99	1.01	1.37	0.38	1.03
Refractory (Fig 4C)	7.03	0.76	0.54	0.32	0.77
Models	ρ	σ_{on}	σ_{off}	d	
Telegraph (Fig 4B)	9.25	0.35	0.94	1.36	/
Telegraph (Fig 4C)	9.67	0.31	1.34	0.97	/

[1] A. Raj, C. S. Peskin, D. Tranchina, D. Y. Vargas, and S. Tyagi, "Stochastic mRNA synthesis in mammalian cells," *PLoS Biology*, vol. 4, no. 10, p. e309, 2006.

[2] S. Iyer-Biswas, F. Hayot, and C. Jayaprakash, "Stochasticity of gene products from transcriptional pulsing," *Physical Review E—Statistical, Nonlinear, and Soft Matter Physics*, vol. 79, no. 3, p. 031911, 2009.

[3] R. Grima, D. R. Schmidt, and T. J. Newman, "Steady-state fluctuations of a genetic feedback loop: An exact solution," *The Journal of Chemical Physics*, vol. 137, no. 3, 2012.

[4] Z. Cao, T. Filatova, D. A. Oyarzún, and R. Grima, "A stochastic model of gene expression with polymerase recruitment and pause release," *Biophysical Journal*, vol. 119, no. 5, pp. 1002–1014, 2020.

[5] N. Kumar, T. Platini, and R. V. Kulkarni, "Exact distributions for stochastic gene expression models with bursting and feedback," *Physical Review Letters*, vol. 113, no. 26, p. 268105, 2014.

[6] Y. Wang, Z. Yu, R. Grima, and Z. Cao, "Exact solution of a three-stage model of stochastic gene expression including cell-cycle dynamics," *The Journal of Chemical Physics*, vol. 159, no. 22, 2023.

[7] Q. Jiang, X. Fu, S. Yan, R. Li, W. Du, Z. Cao, F. Qian, and R. Grima, "Neural network aided approximation and parameter inference of non-markovian models of gene expression," *Nature Communications*, vol. 12, no. 1, p. 2618, 2021.

[8] Y. Wang, J. Szavits-Nossan, Z. Cao, and R. Grima, "Joint distribution of nuclear and cytoplasmic mRNA levels in stochastic models of gene expression: analytical results and parameter inference," *Physical Review Letters*, vol. 135, no. 6, p. 068401, 2025.

[9] Z. Cao and R. Grima, "Analytical distributions for detailed models of stochastic gene expression in eukaryotic cells," *Proceedings of the National Academy of Sciences*, vol. 117, no. 9, pp. 4682–4692, 2020.

[10] ——, "Linear mapping approximation of gene regulatory networks with stochastic dynamics," *Nature Communications*, vol. 9, no. 1, p. 3305, 2018.

[11] C. Jia and R. Grima, "Holimap: an accurate and efficient method for solving stochastic gene network dynamics," *Nature Communications*, vol. 15, no. 1, p. 6557, 2024.

[12] (2025) Nist digital library of mathematical functions. National Institute of Standards and Technology. Release 1.2.1 of 2025-03-15. [Online]. Available: https://dlmf.nist.gov/

[13] E. L. Ince, *Ordinary Differential Equations.* Dover Publications, 1956, originally published 1926.
